# Synthesis, Biological Activity and Preliminary in Silico ADMET Screening of Polyamine Conjugates with Bicyclic Systems

**DOI:** 10.3390/molecules22050794

**Published:** 2017-05-12

**Authors:** Marta Szumilak, Malgorzata Galdyszynska, Kamila Dominska, Irena I. Bak-Sypien, Anna Merecz-Sadowska, Andrzej Stanczak, Boleslaw T. Karwowski, Agnieszka W. Piastowska-Ciesielska

**Affiliations:** 1 Department of Hospital Pharmacy, Faculty of Pharmacy, Medical University of Lodz, 1 Muszynskiego Street, 90-151 Lodz, Poland; marta.szumilak@umed.lodz.pl (M.S.); andrzej.stanczak@umed.lodz.pl (A.S.); 2 Department of Comparative Endocrinology, Medical University of Lodz, 7/9 Zeligowskiego Street, 90-752 Lodz, Poland; malgorzata.galdyszynska@gmail.com (M.G.); kamila.dominska@umed.lodz.pl (K.D.); agnieszka.piastowska@umed.lodz.pl (A.W.P.-C.); 3 Food Science Department, Faculty of Pharmacy, Medical University of Lodz, 1 Muszynskiego Street, 90-151 Lodz, Poland; irena.bak-sypien@umed.lodz.pl (I.I.B.-S.); anna.merecz-sadowska@umed.lodz.pl (A.M.-S.); 4 Laboratory of Cell Cultures and Genomic Analysis, Medical University of Lodz, 7/9 Zeligowskiego Street, Lodz 90-752, Poland

**Keywords:** polyamine conjugates, anticancer activity, DNA binding studies, in silico ADMET screening

## Abstract

Polyamine conjugates with bicyclic terminal groups including quinazoline, naphthalene, quinoline, coumarine and indole have been obtained and their cytotoxic activity against PC–3, DU–145 and MCF–7 cell lines was evaluated in vitro. Their antiproliferative potential differed markedly and depended on both their chemical structure and the type of cancer cell line. Noncovalent DNA-binding properties of the most active compounds have been examined using ds–DNA thermal melting studies and topo I activity assay. The promising biological activity, DNA intercalative binding mode and favorable drug-like properties of bis(naphthalene-2-carboxamides) make them a good lead for further development of potential anticancer drugs.

## 1. Introduction

Bisintercalators are a group of compounds which interact reversibly with the DNA double helix. They were designed in order to overcome the limitations of monointercalators, e.g., undesirable side effects and the development of multidrug resistance [1]. Their chemical structure is characterized by the presence of two planar, polycyclic aromatic systems covalently linked by an aminoalkyl chain of different length and rigidity. Simultaneous insertion of two intercalating systems into a DNA double helix results in higher DNA affinity, slower dissociation kinetics and sequence selectivity in comparison to monointercalating agents [2]. Moreover, their binding capacity to DNA may be increased by groove or phosphate interactions of positively charged polyamine linkers connecting two intercalating moieties [3]. This kind of molecules can be modified within both planar terminal groups and the polyamine linker. Many research groups have been interested in designing various groups of bisintercalating agents [4]. These are usually molecules with extended polyaromatic chromophores such as bisnaphthalimides [5,6,7,8], bisacridines [9,10,11] bisphenazines [12,13,14,15] or bisanthracyclines [5,16,17,18]. Although bicyclic chromophores are common in bisintercalator natural products and their derivatives [19], a literature review revealed limited information on small synthetic molecules with bicyclic terminal moieties which exhibit antiproliferative activity [20] or act via bisintercalative binding mode [21,22]. In our attempt to design new entities with anticancer activity based on the general structural characteristics of bisintercalators we focused on symmetrical compounds with bicyclic terminal moieties. Previously, we reported the synthesis and anticancer activity of dimeric quinoline, cinnoline, phthalimide and chromone derivatives with 1,4-bis(3-aminopropyl)piperazine, 4,9-dioxa-1,12-dodecanediamine, or 3,3′-diamino-*N*-methyldipropylamine as polyamine linkers [23,24]. Some of them, mainly chromone and quinoline derivatives, exhibited promising antiproliferative activity toward the highly aggressive A375 melanoma cell line [23,24] the PC–3 prostate adenocarcinoma cell line, the DU–145 prostate carcinoma cell line and the MCF–7 mammary gland adenocarcinoma cell line [25]. IC_50_ values for the most active compound were in the range of 16.8 to 26.6 µM [23,24,25]. In addition, it was elucidated that bis(4-aminoquinoline-3-carboxamide) derivative with 1,4-bis(3-aminopropyl)piperazine as the linker has the ability to interact with double helix via an intercalative binding mode [26]. On the other hand, the biological activity of dimeric cinnoline derivatives was not satisfactory. The data showed that small changes in the structure of these molecules might have a substantial impact on their biological activity [23]. Therefore, in the current study, we decided to introduce several types of bicyclic terminal groups varying in terms of presence and position of heteroatoms (nitrogen, oxygen) or functional groups, namely quinazoline, naphthalene, quinoline, coumarin (2*H*-chromen-2-one) and indole in order to better understand the relationship between biological activity and chemical structure. When designing the linker, apart from 1,4-bis(3-aminopropyl)piperazine, 4,9-dioxa-1,12-dodecanediamine and 3,3′-diamino-*N*-methyldipropylamine we decided to additionally use polyamine-bis(3-amino-propyl)amine to gain insight into the role of the methyl group present on the central nitrogen atom of 3,3′-diamino-*N*-methyldipropylamine in the biological activity. Newly synthesized compounds were screened in vitro for antiproliferative activity against the prostate adenocarcinoma cell line line PC–3, prostate carcinoma cell line DU–145 and mammary gland adenocarcinoma cell line MCF–7. The DNA binding properties of the most active compounds were evaluated by ds–DNA thermal melting studies and topoisomerase I (topo I) activity assay. Finally, according to the paradigm that parallel optimization of ADMET properties along with synthesis and assessing cytotoxic activity in vitro, offers a greater chance of identifying a high quality future therapeutics [27] preliminary in silico ADMET screening was performed to evaluate the potential of the most active compounds to be qualified as drug candidates.

## 2. Results and Discussion

### 2.1. Chemistry

**Scheme 1:**
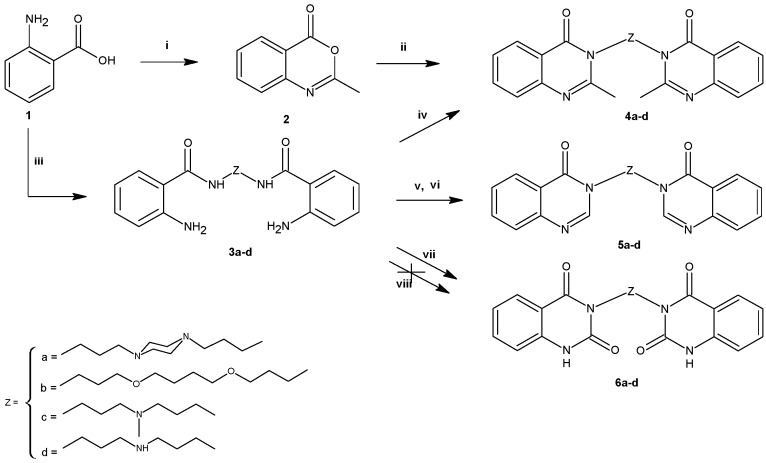
Synthesis of polyamine conjugates with terminal quinazoline moieties. *Reagents and conditions*: (**i**) (CH_3_CO)_2_O, reflux, 3 h; (**ii**) H_2_NZNH_2_, CH_3_CN, reflux, 1 h, NaOH aq, r.t., 24 h; (**iii**) CDI, DMF or CH_3_CN, r.t., 3 h; (**iv**) (CH_3_CO)_2_O, reflux, 3 h; (**v**) HCOOH, 100 °C, 4 h, (**vi**) triethyl orthoformate, CH_3_COOH anhydrous, reflux, 3 h; (**vii**) CDI, DMF, 40 °C, 4 h; (**viii**) oxalyl chloride, toluene, reflux, 6 h.

Compounds **4a**–**d** with quinazoline systems as terminal moieties were prepared by the procedure depicted in Scheme 1. The starting material 2-methyl-4(*H)*-benzoxazin-4-one (**2**) was obtained from anthranilic acid (**1**) by cyclodehydration in acetic anhydride. Its analytical data was in agreement with literature values [28]. Anthranilic acid (**1**) reacted with polyamines **a**–**d** in the presence of 1,1’-carbonyldiimidazole (CDI) to give bisanthranilamides **3a**–**d**, as confirmed by the presence of a characteristic amide signal at 8.2 ppm (DMSO-*d*_6_) or 7.1 ppm (CDCl_3_) in their corresponding ^1^H-NMR spectra [29].

Two synthetic pathways led to 2-methylquinazolin-4(3*H*)-one derivatives **4a**–**d** (Scheme 1): via conversion of the internal amidine salts formed in the reaction of **2** with 0.5 equiv. of an appropriate polyamine **a**–**d** according to the mechanism described by Errede et al. and Stanczak et al. [30,31,32] or direct formation from bisanthranilamides **3a**–**d** in an excess of acetic anhydride. ^1^H-NMR spectra of the final compounds **4a**–**d** indicated a lack of the characteristic signal of the NH proton of an amide group, present in the ^1^H-NMR spectra of **3a**–**d** at 8.2 ppm (DMSO-*d*_6_) or 7.1 ppm (CDCl_3_), in agreement with the expected structures.

Synthesis of compounds **5a**–**d** was achieved by allowing bisanthranilamides **3a**–**d** to undergo ring closure either with triethyl orthoformate [33] or formic acid [34] under reflux (Scheme 1). Their ^1^H-NMR spectra showed in each case the characteristic singlet at ~8.3 ppm assigned to the proton of a –N=CH- group.

The treatment of bisanthranilamides **3a**–**d** with oxalyl chloride, according to the method described by Malamas et al., [35] did not give the desired compounds **6a**–**d**. Therefore, they were obtained by cyclization of **3a**–**d** with CDI [36]. Their ^1^H-NMR spectra exhibited a singlet at about 11.4 ppm due to the NH proton of the quinazoline-2,4(1*H*,3*H*)-dione moiety, while the signals of the NH proton of bisanthranilamides **3a**–**d**, present in the aromatic region, were not observed.

**Scheme 2:**
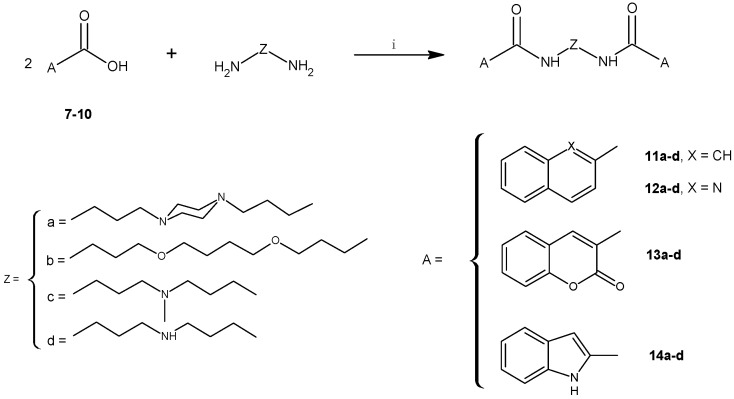
Synthesis of polyamine conjugates with naphthalene **11**, quinoline **12**, coumarin **13** and indole **14** as terminal scaffolds. *Reagents and conditions*: (**i**) CDI, DMF or CH_3_CN, rt, 3 h.

Biscarboxamides with two naphthalene **11**, quinoline **12**, coumarin **13**, indole **14** moieties were typically formed from 0.5 equiv. of appropriate polyamine **a**–**d** and carboxylic acids **7**–**10** in the presence of CDI, according to the method described earlier (Scheme 2) [29]. The formation of the final products was confirmed by the presence of characterisitic amide signals at 8.5–8.6 ppm for **11**, 8.7–9.1 ppm for **12**, 8.4–8.5 ppm for **13**, and 8.7–9.0 ppm for **14** in the obtained ^1^H-NMR spectra.

For biological experiments, compounds **4a**, **4d**, **5a**, **5c**, **6a**, **6c**, **11a**, **12d**, **13a**, **13c**, **13d**, **14a**, **14b**, **14d** were converted into the corresponding hydrochloride/hydrobromide by dissolving the corresponding base in absolute ethanol and treating with dry diethyl ether saturated with HCl or HBr.

### 2.2. Biological In Vitro Evaluation

The anticancer potential of the newly synthesized polyamine conjugates with bicyclic terminal groups was assessed in three cancer cell lines, namely the prostate adenocarcinoma cell line PC–3, prostate carcinoma cell line DU–145 and mammary gland adenocarcinoma cell line MCF–7 using standard WST–1 assays. This assay is based on the cleavage of the water-soluble tetrazolium salt WST–1 to formazan catalysed by cellular mitochondrial dehydrogenases [37]. The amount of formazan dye obtained directly correlates with the number of live cells in a culture.

Our previous observations indicated that polyamine derivatives with chromone and quinoline as terminal moieties had the potential to attenuate proliferation in melanoma, two prostate and one breast cancer cell lines [23,24,25]. In attempt to gain insight into the structure-activity relationships in this group of symmetrical molecules, compounds with different bicyclic terminal systems were designed. Results of the presented experiments revealed significant differences in their anticancer activity (Table 1).

**Figure 1:**
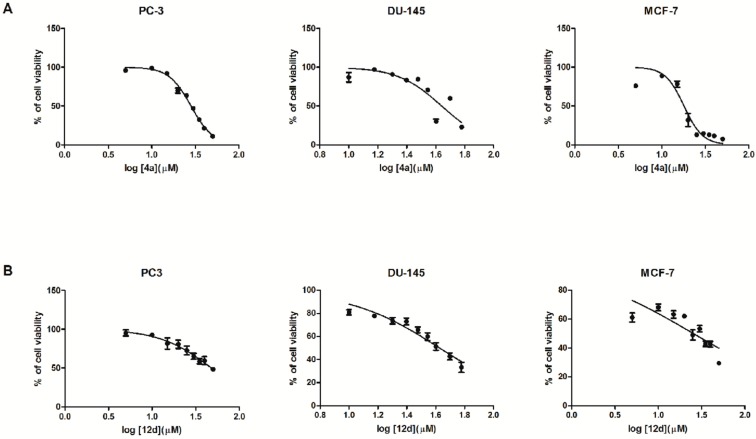
Dose response curves. PC–3, DU–145 and MCF–7 cells were exposed to either **4a** (**A**) or **12d** (**B**) for 48 h, followed by the WST–1 assay to determine cell viability (mean ± SD).

The analysis of results obtained for **4a**–**d**, **5a**–**d**, **6a**–**d** demonstrated that only bis(2-methylquinazolin-4(3*H*)-one) with 1,4-bis(3-aminopropyl)piperazine as the linker (compound **4a**) exhibited cytotoxicity toward prostate and breast cancer cells (Figure 1A).

The antiproliferative activity of **4a** depended upon the cell line, what was expressed by the corresponding IC_50_ values: 17.95 μM, 28.24 μM, 43.63 μM for MCF–7, PC–3 and DU–145 cell lines, respectively. The rest of the investigated bisquinazoline derivatives were biologically inactive or their poor solubility (e.g., in case of the bis(quinazoline-2,4(1*H*,3*H*)-dione) derivatives **6a**–**d**) precluded an assessment of the biological activity.

Conversion of the terminal chromone systems to coumarin moieties did not result in increased biological activity. Biscoumarin derivatives **13a**–**d** were essentially inactive under the experimental conditions. As far as bis(quinoline-2-carboxamides) **12a**–**d** were concerned, only **12d** with bis(3-aminopropyl)amine as the linker exhibited moderate antiproliferative potency (Figure 1B) expressed in IC_50_ values: 25.45 μM, 42.63 μM and 48.08 μM for MCF–7, DU–145 and PC–3 cells, respectively.

It was demonstrated that bis(quinoline-2-carboxamides) **12a**–**d** had reduced activity in comparison to bis(4-aminoquinoline-3-carboxamide) derivatives described earlier [23], which might prove that the substitution of amino group at the 4–position of quinoline system and changing the position of the linker attachment can have significant impact on antiproliferative potential of bisquinoline derivatives.

**Figure 2:**
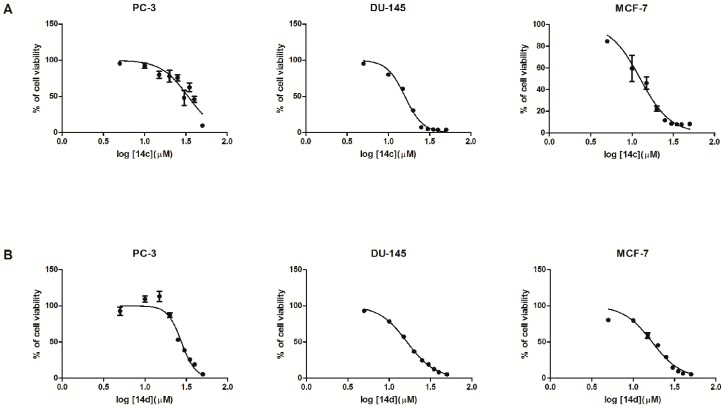
Dose response curves. PC–3, DU–145 and MCF–7 cells were exposed to either **14c** (**A**) or **14d** (**B**) for 48 h, followed by the WST–1 assay to determine cell viability (mean ± SD).

Moreover, it could be observed that “the size” of the terminal moiety plays an important role in the biological activity. Changing a six-membered ring (pyridine) in the quinoline system for a five-membered ring (pyrrole) in the indole system resulted in improved potency, what was illustrated by the following IC_50_ values: 15.51 μM (MCF–7 cells), 15.86 μM (DU–145 cells) and 35.72 μM (PC–3 cells) for bis(indole-2-carboxamide) with 3,3′-diamino-*N*-methyldipropylamine as the linker (compound **14c**) and 16.46 μM (DU–145 cells), 16.91 μM (MCF–7 cells) and 27.59 μM (PC–3 cells) for **14d** where the indole moieties were connected by bis(3-aminopropyl)amine (**d**) (Figure 2A,B).

**Figure 3:**
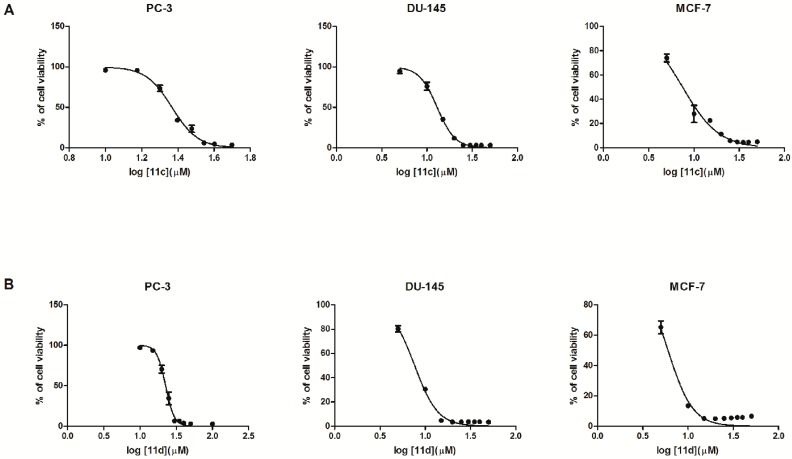
Dose response curves. PC–3, DU–145 and MCF–7 cells were exposed to either **11c** (**A**) or **11d** (**B**) for 48 h, followed by the WST–1 assay to determine cell viability (mean ± SD).

Among all tested compounds, bis(naphthalene-2-carboxamide) with 3,3′-diamino-*N*-methyldipropylamine and bis(3-aminopropyl)amine as linkers (compounds **11c**, **11d**, respectively) exhibited a substantial influence on the proliferation of breast cancer cells and both prostate cell lines (Figure 3A,B). Moreover, compounds **11c** and **11d** caused 50% growth inhibition (IC_50_) at lower concentration in mammary gland adenocarcinoma cells (7.48 μM and 6.00 μM, respectively), in comparison to prostate adenocarcinoma (23.30 μM and 22.57 μM, respectively). This data might be an evidence that the presence of heteroatoms in terminal moieties is not essential for antiproliferative activity.

The obtained results also showed the crucial role of the linker in the compounds’ biological activity. As presented in Table 1, the majority of the investigated molecules exhibiting anticancer activity had 3,3′-diamino-*N*-methyldipropylamine (**c**) or bis(3-aminopropyl)amine (**d**) as the linker (Scheme 1 and Scheme 2). It is worth noting that in case of naphthalene derivatives **11c** and **11d** removing the methyl group from the central nitrogen atom of 3,3′-diamino-*N*-methyldipropylamine (**c**) led to a slight decrease in IC_50_ values. Molecules containing 4,9-dioxa-1,12-dodecanediamine (**b**) as a spacer, regardless the type of terminal moiety, were inactive toward the three used cell lines.

### 2.3. DNA Interaction Studies

Since the synthesized compounds exhibited noticeable differences in biological activity, we chose only compounds **4a**, **11c**, **11d**, **12d**, **14c**, **14d** with the highest antiproliferative activity against PC–3, DU–145 and MCF–7 cells to perform a preliminary assessment of their ds–DNA binding mode by ds–DNA thermal melting studies and topoisomerase I activity assay.

#### 2.3.1. Thermal Melting Studies

The ds–DNA binding ability of compounds **4a**, **11c**, **11d**, **12d**, **14c**, **14d** was initially evaluated by thermal stability studies according to method reported by Guedin et al. [38]. The examined polyamine derivatives were tested at a concentration of 15 μM after oligonucleotide strand hybridization (Table 2). The following complementary 29-mer oligonucleotides i.e., 5’-AAA TTA ATA TGT ATT GTA TAT AAA TTA TT-3’ and 3’-TTT AAT TAT ACA TAA CAT ATA TTT AAT AA-5’ were employed. Non self-complementary base sequences have been chosen in order to avoid the influence of unspecific interaction between oligonucleotides and the investigated compounds on the intercalation process. Moreover, both strands of ds-DNA contained relevant amounts of purine and pyrimidine bases. Double stranded oligonucleotide without the tested compounds was used as a negative control and the well-known intercalator 9-aminoacridine **9AA** (100 μM) was employed as a positive one.

The analysis of *T*_m_ values has shown that only compounds **11c** and **11d** exhibited the ability to increase ds–DNA stability by 3°C and 6°C in comparison to the reference ds-oligonucleotide, respectively. In contrast, for other discussed compounds **4a**, **12d**, **14c**, **14d** the melting temperature changes were negligible (Table 2). It is worth noting that for reference compound **9AA**, *T*_m_ = 78 °C was denoted. Based on the above results, it may be postulated that **11c** and **11d** have the ability to stabilize the double helix. Therefore, the topoisomerase I activity assay was used to more definitively establish the nature of interactions between **11c**, **11d** and ds–DNA.

#### 2.3.2. Topoisomerase I Activity Assay

**Figure 4:**
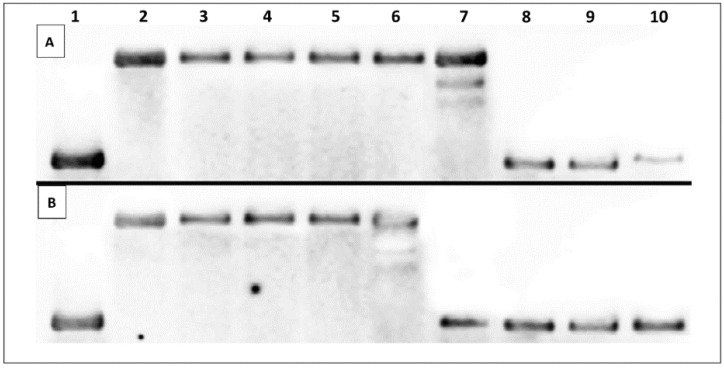
Influence of compounds **11c** (**A**), **11d** (**B**) on conversion of relaxed plasmid DNA to supercoiled molecule. Control reactions were carried out in the absence of topo I (supercoiled plasmid) (lane 1), with topo I (relaxed plasmid) (lane 2), with topo I and 0.1% DMSO (lane 3). Plasmid conformation was analyzed in increasing concentrations of investigated compounds (lane 4–9, concentration: 1; 5; 10; 15; 20 and 30 μM, respectively) with constant topo I concentration. 9AA (100 μM) was used as a positive control (lane 10).

Topoisomerase I activity assay is typically performed to evaluate compounds for their ability to intercalate into DNA. It allows one to differentiate intercalators from topoisomerase inhibitors. Relaxed plasmid treated with topo I and intercalative agent is converted into a negatively supercoiled form, whereas in the presence of a topo I inhibitor the relaxation process can be still observed [39]. Our previous study clearly indicated that polyamine conjugates with bicyclic systems such as quinoline have the ability to interact with ds-DNA via intercalative binding mode [26]. The present experiments showed that compounds **11c** and **11d** with naphthalene as terminal moiety exhibit similar properties. As can be seen in Figure 4, supercoiled DNA is fully relaxed in the presence of topo I and in the absence of the drug. It has been observed that topo I in the presence of compound **11c** at the concentration >10 μM and compound **11d** at the concentration >15 μM converted relaxed plasmid to supercoiled molecule.

Based on thermal melting studies and topo I activity assay it can be postulated that only **11c** and **11d** exhibit stacking interactions with double helix DNA. This may indicate that the presence of a naphthalene moiety together with 3,3′-diamino-*N*-methyldipropylamine (**c**) or bis(3-aminopropyl)-amine (**d**) as linkers is crucial for the assumed binding mode (Scheme 2).

### 2.4. Preliminary In Silico ADME Screening

In accordance with modern trends in drug discovery involving parallel evaluation of efficacy and ADMET properties of drug candidates at the earliest stages in their development [40], computer-aided ADMET screening was performed for compounds **4a**, **11c**, **11d**, **12d**, **14c**, **14d** exhibiting the highest activity against PC–3, DU–145 and MCF–7 cell lines. From the many available software tools for predicting ADMET properties, ACD/Percepta obtained from Advanced Chemistry Development, Inc. (ACD/Labs) was chosen [41]. *Drug-likeness* of the examined compounds was evaluated using the Drug Profiler Module. ADMET properties were calculated using the ADME and Toxicity Modules. Results are presented in Table 3 and Table 4, respectively. All calculations were solely based on the chemical structure of molecules.

In the ACD/Percepta software, *Lipinski's Rule of Five* (RO5) for oral bioavailability involving hydrogen bond acceptors (HBA), hydrogen bond donors (HBD), molecular weight (M_w_) and the logarithm value of octanol/water partition coefficient (log*P*) descriptors was employed to assess drug-likeness of compounds [42]. According to the predictions, all tested molecules showed good drug-likeness compliance (Table 3) which might indicate that these compounds could be further developed as oral drug candidates.

Moreover, the results disclosed in Table 4 show that all tested compounds exhibited %HIA > 70% and were considered as highly absorbed with almost 100% contribution of transcellular route to absorption. However, there were noticeable differences in estimated jejunal permeability coefficients at pH 6.5 (*P_e_*) and absorption rate constants (*k*_a_) which were the highest for **4a**, **11c** and **11d**. This might suggest that these compounds can be more efficiently absorbed in the human intestine.

The brain delivery potential of the examined compounds was assessed according to the modelling approach assuming that combination of parameters corresponding to brain/plasma equilibration rate (log(PS*fu, brain)) and the extent of brain penetration at equilibrium (log*BB*) allows proper estimation of central nervous system (CNS) exposure [43]. Only **4a** was described as a compound sufficiently accessible to the CNS to exhibit pharmacological effects in the brain (Score ˃ −3). The rest of biologically active compounds were denoted as non-penetrants (Score ≤ −3.5). This might indicate that possible CNS adverse effects could be low or absent, but on the other hand, it could be a limiting factor in the therapy of brain tumors.

As far as plasma protein binding (PPB) is concerned, the majority of analysed compounds, namely: **11c**, **11d**, **12d**, **14c**, **14d**, were likely to be extensively bound to plasma proteins (%PPB > 90%). This property is not desirable due to loss of “active molecule” efficacy, since it is usually a free fraction of drug that is responsible for the pharmacological activity [44]. From all tested compounds, **11c** exhibited the highest human serum albumin affinity constant (Table 4). Only **4a** was categorized as moderately bound to plasma protein (40% < %PPB ≤ 80%) with the lowest affinity constant to human serum albumin.

The assessment of acute toxicity is a very important stage, indicating the potential safety of future drugs. Computational prediction of toxicity parameters is a useful tool helping to rationalize time and financial costs of in vitro testing on animals [45]. On the basis of calculated LD_50_ (oral administration to rats) values the evaluated compounds have been allocated to the different categories defined by the OECD [46]. The most probable OECD hazard category for **4a** is III (toxic if swallowed), and for the rest of compounds **11c**, **11d**, **12d**, **14c**, **14d** it is IV (harmful if swallowed) which is not surprising for potential anticancer drugs.

## 3. Materials and Methods

### 3.1. General Information

Reagents and solvents were purchased from common commercial suppliers. Melting points were measured on an Electrothermal apparatus (Barnstead International, Dubuque, IA, USA) in open capillaries and are uncorrected. Compounds were purified by column chromatography over silica gel (Kieselgel 60, 0.060–0.2 mm, Merck, Sigma-Aldrich, Saint Louis, MO, USA) or by crystallization from appropriate solvents. Elemental analyses were carried out with a Series II CHNS/O Analyzer 2400 (Perkin Elmer, Waltham, MA, USA) and were within ±0.4% of the theoretical values. ^1^H-NMR and ^13^C-NMR spectra were recorded on a Mercury 300 MHz (Varian Inc. currently Agilent Technologies, Palo Alto, CA, USA) or Avance III 600 MHz spectrophotometer (Bruker Company, Billerica, MA, USA) in CDCl_3_ or DMSO-*d*_6_ solutions with TMS as an internal standard. The spectra data of new compounds refer to their free bases. Chemical shifts were given in δ (ppm) and the coupling constants *J* in Hertz (Hz). The following abbreviations were used to describe peak patterns when appropriate: s (singlet), d (doublet), t (triplet), q (quartet), quin (quintet), m (multiplet), brs (broad singlet).

### 3.2. Chemistry

#### 3.2.1. General Procedure for the Synthesis of **3a**–**d**

Compound **1** (10 mmol) and CDI (11 mmol) in DMF (100 mL) were stirred for 1 h at room temperature. Then the appropriate polyamine (**a**–**d**) (6 mmol) was added and stirring was continued for additional 2 h. At the end of the reaction, the mixture was filtered. The solvent was removed in vacuum. 20 mL of H_2_O was added to the residue (compounds **3a**,**b**) and left for 24 h at 5 °C. Then the solid was filtered off, washed with H_2_O and crystallized from DMF/H_2_O. The residue was purified by column chromatography over silica gel (CHCl_3_/MeOH, 100:1–0:1, *v*/*v*) to give compounds **3c**,**d**.

*N,N'-[Piperazine-1,4-diylbis(propane-3,1-diyl)]bis(2-aminobenzamide)* (**3a**). White solid. Yield 83.7%; M.p. 209.9–211.8 °C; ^1^H-NMR (300 MHz, DMSO-*d*_6_) δ_H_: 8.27 (t, 2H, *J* = 5.1 Hz, 2 × C(O)N*H*), 7.44 (d, 2H, *J* = 7.8 Hz, *H_arom_*_._), 7.11 (t, 2H, *J* = 8.2 Hz, *H_arom._*), 6.67 (d, 2H, *J* = 8.2 Hz, *H_arom._*), 6.49 (t, 2H, *J* = 7.8 Hz, *H_arom_*_._), 6.37 (brs, 4H, 2 × N*H*_2_), 3.23 (q, 4H, *J* = 6.6 Hz, 2 × (C(O)N-C*H*_2_)), 2.56–2.24 (cluster, 12H, 8 × *H_piperazine_* and 2 × N-C*H*_2_), 1.65 (quin, 4H, *J* = 6.6 Hz, 2 × C(O)NCH_2_C*H*_2_CH_2_N) ppm; ^13^C-NMR (75 MHz, DMSO-*d*_6_) δ_C_: 168.46 (C(O)), 149.27 (C-NH_2_), 131.28, 127.76, 116.12, 114.78, 114.35, 56.01 (4 × C*_piperazine_*), 52,83 (C-N*_amide_*), 37.76 (C-N*_piperazine_*), 26.00 ppm; Anal. Calcd. (%) for C_24_H_34_N_6_O_2_: C 65.73; H 7.81; N 19.16. Found (%): C, 65.35; H, 8.05; N, 19.46.

*N,N'-[(Butane-1,4-diylbis(oxy))bis(propane-3,1-diyl)]bis(2-aminobenzamide)* (**3b**). White solid. Yield 60.0%; M.p. 107.6–109.0 °C; ^1^H-NMR (300 MHz, DMSO-*d*_6_) δ_H_: 8.16 (t, 2H, *J* = 5.4 Hz, 2 × C(O)N*H*), 7.44 (d, 2H, *J* = 7.8 Hz, *H_arom._*), 7.11 (dd, 2H, *J* = 8.2, 8.2 Hz, *H_arom._*), 6.67 (d, 2H, *J* = 8.2 Hz, *H_arom._*), 6.49 (dd, 2H, *J* = 7.8 Hz, *H_arom._*), 6,36 (brs, 4H, 2 × N*H*_2_), 3.45–3.19 (cluster, 12H, 2 × C(O)NC*H*_2_ and 4 × C*H*_2_O), 1.81–1.65 (m, 4H, 2 × OCH_2_C*H*_2_CH_2_N), 1.6–1.25 (m, 4H, OCH_2_C*H*_2_C*H*_2_CH_2_O) ppm; ^13^C-NMR (75 MHz, DMSO-*d*_6_) δ_C_: 168.43 (C(O)), 149.31 (C-NH_2_), 131.29, 127.78, 116.14, 114.79, 114.36, 70,7 (C-O*_but._*), 67.74 (C-O*_prop_*.), 44.51 (C-N*_amide_*), 28.86, 26.38 ppm; Anal. Calcd. (%) for C_24_H_34_N_4_O_4_: C 65.14; H 7.74; N 12.66. Found (%): C, 65.50; H, 7.80; N, 12.45.

*N,N'-[(Methylazanediyl)bis(propane-3,1-diyl)]bis(2-aminobenzamide)* (**3c**). Yellow oil. Yield 59.6%; ^1^H-NMR (300 MHz, CDCl_3_) δ_H_: 7.26 (dd, 2H, *J* = 7.8, 1.5 Hz, *H_arom._*), 7.17 (ddd, 2H, *J* = 7.2, 7.2, 1.5 Hz, *H_arom._*), 7.09 (brs, 2H, 2 × C(O)N*H*), 6.67–6.57 (m, 4H, *H_arom._*), 5.51 (brs, 4H, 2 × N*H*_2_), 3.48–3.38 (m, 4H, 2 × C(O)NC*H*_2_), 2.52–2.44 (m, 4H, 2 × C*H*_2_N(CH_3_)), 2.28 (s, 3H, NC*H*_3_), 1.74 (quin, 4H, *J* = 6.5 Hz, CH_2_C*H*_2_CH_2_) ppm; ^13^C-NMR (75 MHz, CDCl_3_) δ_C_: 168.49 (C(O)), 149.24 (C-NH_2_), 131.22, 127.70, 116.10, 114.90, 114.38, 55.62 (C-N), 45.05 (N-CH_3_), 41.61 (C-N*_amide_*), 26.50 ppm; Anal. Calcd. (%) for C_21_H_29_N_5_O_2_: C 65.77; H 7.62; N 18.26. Found (%): C, 65.50; H, 7.80; N, 18.45.

*N,N'-[Azanediylbis(propane-3,1-diyl)]bis(2-aminobenzamide)* (**3d**). Yellow oil. Yield 65.6%; ^1^H-NMR (600 MHz, CDCl_3_) δ_H_: 7.33 (dd, 2H, *J* = 7.9, 1.2 Hz, *H_arom_*_._), 7.21–7.16 (m, 4H, *H_arom_*_._ and 2C(O)N*H*), 6.68 (dd, 2H, *J* = 8.2, 1.0 Hz, *H_arom_*_._), 6.63 (ddd, 2H, *J* = 7.9, 6,1, 1.0 Hz, *H_arom_*_._), 5.53 (brs, 4H, 2 × N*H*_2_), 3.51 (ddd, 4H, *J* = 6.0, 6.0, 6.0 Hz, 2 ×NC*H*_2_), 2.77 (t, *J* = 6.4 Hz, 4H, 2 × C*H*_2_N(CH_3_)), 1.85–1.71 (m, 4H, 2 × CH_2_C*H*_2_CH_2_), 1.56 (brs, 1H, N*H*) ppm; ^13^C-NMR (75 MHz, CDCl_3_) δ_C_: 168.45 (C(O)), 149.20 (C-NH_2_), 131.19, 127.69, 116.11, 114.92, 114.35, 45.00 (C-N), 42.00 (C-N*_amide_*), 26.49 ppm; Anal. Calcd. (%) for C_20_H_27_N_5_O_2_: C 65.02; H 7.37; N 18.96. Found (%): C, 65.40; H, 7.80; N, 18.66.

#### 3.2.2. General Procedure for the Synthesis of **4a**–**d**

Method A: A mixture of **2** (10 mmol) and the appropriate polyamine **a**–**d** (6 mmol) in CH_3_CN (100 mL) was refluxed for 1 h and then left for 12 h at room temperature. The crude product was filtered off, dried and dissolved at room temperature in a minimum amount of 2% NaOH and then diluted two-fold. The clear solution became cloudy within 1 h and precipitation was complete within 24 h. The product was collected by filtration and recrystallized from EtOH/Et_2_O.

Method B: Bisanthranilamide **3a**–**d** (10 mmol) in acetic anhydride (15 mL) was refluxed for 2 h. After cooling, the solvent was removed under vacuum. The residue was dissolved in water and made alkaline with 10% NH_4_OH to obtain the free base. The precipitate formed was filtered off, washed with H_2_O and crystallized from EtOH/Et_2_O.

*3,3'-[Piperazine-1,4-diylbis(propane-3,1-diyl)]bis(2-methylquinazolin-4(3H)-one)* (**4a**). White solid. Yield 40.5%; M.p. 212.8–213.5 °C; ^1^H-NMR (300 MHz, CDCl_3_) δ_H_: 8.23 (dd, 2H, *J* = 8.0, 1.5 Hz, C*H_arom_*_._), 7.71 (ddd, 2H, *J* = 8.3, 7.0, 1.5 Hz, C*H_arom_*_._), 7.60 (d, 2H, *J* = 8.3 Hz, C*H_arom_*_._), 7.43 (ddd, 2H, *J* = 8.0, 7.0, 1.2 Hz, C*H_arom_*_._), 4.23–4.05 (m, 4H, 2 × NC*H*_2_), 2.67 (s, 6H, 2 × C*H*_3_), 2.58–2.20 (cluster, 12H, 4 × C*H*_2*piperazine*_ and 2 × C*H*_2_N), 2.00–1.84 (m, 4H, 2 × CH_2_C*H*_2_CH_2_) ppm; ^13^C-NMR (75 MHz, CDCl_3_) δ_C_: 162.12 (C(O)), 154.13 (C=N), 147.37, 134.44, 134.02, 126.94, 126.40, 126.4, 55.50 (s, 4 × C*_piperazine_*), 44.36 (C-N*_amide_*), 43.37 (C-N*_piperazine_*), 25.79, 23.46 (CH_3_) ppm; Anal. Calcd. (%) for C_28_H_34_N_6_O_2_ × 2HCl × 6H_2_O: C, 50.45; H, 7.01; N, 12.60. Found (%): C, 50.32; H, 6.80; N, 12.43.

*3,3'-[(Butane-1,4-diylbis(oxy))bis(propane-3,1-diyl)]bis(2-methylquinazolin-4(3H)-one)* (**4b**). White solid*.* Yield 52.9%; M.p. 72.8–74.5 °C; ^1^H-NMR (300 MHz, CDCl_3_) δ_H_: 8.23 (dd, 2H, *J* = 8.0, 1.5 Hz, C*H_arom_*_._), 7.71 (ddd, 2H, *J* = 8.3, 7.0, 1.5 Hz, C*H_arom_*_._), 7.60 (d, 2H, *J* = 8.3 Hz, C*H_arom_*_._), 7.42 (ddd, 2H, *J* = 8.0, 7.0, 1.5 Hz, C*H_arom_*_._), 4.28–4.11 (m, 4H, 2 × NC*H*_2_), 3.50 (t, 4H, *J* = 5.8 Hz, 2 × NC*H*_2_), 3.45–3.41 (m, 8H, 4 × C*H*_2_O), 2.67 (s, 6H, 2 × C*H*_3_), 2.12–1.96 (m, 4H, 2 × OCH_2_C*H*_2_CH_2_N), 1.66–1.55 (m, 4H, OCH_2_C*H*_2_C*H*_2_CH_2_O) ppm; ^13^C-NMR (75 MHz, CDCl_3_) δ_C_: 162.14 (C(O)), 154.46 (C=N), 147.35, 134.46, 126.77, 126.54, 126.29, 120.50, 71.25 (O-C*_but_*_._), 67.93 (C*_prop_*_._-O), 42.58 (C-N*_amide_*), 29.34, 26.74, 23.47 (CH_3_) ppm; Anal. Calcd. (%) for C_28_H_34_N_4_O_4_ × ^1^/_2_ H_2_O: C, 67.86; H, 6.30; N, 11.30. Found (%): C, 68.08; H, 6.60; N, 11.36.

*3,3'-[(Methylazanediyl)bis(propane-3,1-diyl)]bis(2-methylquinazolin-4(3H)-one)* (**4c**). White needles*.* Yield 51.6%; M.p. 114.8–116.0 °C; ^1^H-NMR (300 MHz, CDCl_3_) δ_H_: 8.23 (d, 2H, *J* = 8.0 Hz, C*H_arom_*_._), 7.73 (ddd, 2H, *J* = 8.0, 7.0, 1.0 Hz, C*H_arom_*_._), 7.60 (d, 2H, *J* = 8.0 Hz, C*H_arom_*_._), 7.42 (dd, 2H, *J* = 8.0, 7.0 Hz, C*H_arom_*_._), 4.22–4.09 (m, 4H, 2 × NC*H*_2_), 2.68 (s, 6H, 2 × CH_3_), 2.51 (t, *J* = 6.7 Hz, 4H, 2 × C*H*_2_N(CH_3_)), 2.29 (s, 3H, NC*H*_3_), 2.00–1.84 (m, 4H, 2 × CH_2_C*H*_2_CH_2_) ppm; ^13^C-NMR (75 MHz, CDCl_3_) δ_C_: 162.07 (C(O)), 154.22 (C=N), 147.37, 134.22, 126.77, 126.79, 126.43, 120.63, 55.24 (C-N), 43.38 (N-CH_3_), 42.04 (C-N*_amide_*), 29.57, 23.39 (CH_3_) ppm; Anal. Calcd. (%) for C_25_H_29_N_5_O_2_: C 69.58; H 6.77; N 16.22. Found (%): C, 69.19; H, 6.85; N, 16.13.

*3,3'-[Azanediylbis(propane-3,1-diyl)]bis(2-methylquinazolin-4(3H)-one)* (**4d**). White needles. Yield 62.50%; M.p. 277.3 °C with decomp.; ^1^H-NMR (300 MHz, CDCl_3_) δ_H_: 8.24 (dd, 2H, *J* = 8.0, 1.0 Hz, C*H_arom_*_._), 7.72 (ddd, 2H, *J* = 8.0, 7.0, 1.0 Hz, C*H_arom_*_._), 7.62 (d, 2H, *J* = 8.0 Hz, C*H_arom_*_._), 7.44 (dd, 2H, *J* = 8.0, 7.0 Hz, C*H_arom_*_._), 4.24 (dd, 4H, *J* = 7.2, 7.2 Hz, 2 × NC*H*_2_), 2.75 (t, 4H, *J* = 6.6, 6.6 Hz, 2 × C*H*_2_N(CH_3_), 2.69 (s, 6H, 2 × CH_3_), 2.03–1.89 (m, 4H, 2 × CH_2_C*H*_2_CH_2_), 1.65 (brs, 2H, N*H*_2_) ppm; ^13^C-NMR (75 MHz, CDCl_3_) δ_C_: 162.07 (C(O)), 154.22 (C=N), 147.37, 134.22, 126.77, 126.79, 126.43, 120.63, 54.24 (C-N), 42.38 (C-N*_amide_*), 32.04, 23.57 (CH_3_) ppm; Anal. Calcd. (%) for C_24_H_27_N_5_O_2_ × 2HCl: C, 58.78; H 5.96; N 14.28. Found (%): C, 59.10; H, 5.70; N, 14.58.

#### 3.2.3. General Procedure for the Synthesis of **5a**–**d**

Method A: Bisanthranilamide **3a**–**d** (10 mmol) and triethyl orthoacetate (80 mmol) were refluxed for 3 h in anhydrous acetic acid (15 mL). After cooling, the solvent was removed under vacuum. The residue was dissolved in water and made alkaline with 10% NH_4_OH to obtain the free base which was washed with H_2_O and purified by column chromatography over silica gel (CHCl_3_/MeOH, 100:1–10:1, *v*/*v*).

Method B: Bisanthranilamide **3a**–**d** (10 mmol) in 98% formic acid (100 mL) was heated at reflux for 6 h. The solvent was removed under vacuum, and the remaining traces of formic acid were removed by azeotropic evaporation with toluene to give a solid which was recrystallized from DMF/H_2_O (**5a**, **5b**) or EtOH/Et_2_O (**5d**). Compound **5c** was purified by column chromatography over silica gel (chloroform/MeOH, 100:1–10:1, *v*/*v*) to give a yellow oil.

*3,3'-[Piperazine-1,4-diylbis(propane-3,1-diyl)]bis(quinazolin-4(3H)-one)* (**5a**). White solid. Yield 51.0%; M.p. 183.0–184.5 °C; ^1^H-NMR (600 MHz, DMSO-*d*_6_) δ_H_: 8.34 (s, 2H, N=C*H*), 8.16 (dd, 2H, *J* = 7.8, 1.2 Hz, *H_arom_*_._), 7.80 (ddd, 2H, *J* = 8.2, 7.2, 1.5 Hz, *H_arom._*), 7.66 (d, 2H, *J* = 8.2, Hz, *H_arom_*_._), 7.53 (ddd, 2H, *J* = 7.8, 7.2, 1.1 Hz, *H_arom_*_._), 3.99 (dd, 4H, *J* = 6.6, 6.6 Hz, 2 × (N-C*H*_2_)), 2.30–2.19 (cluster, 12H, *H_piperazine_*, 2 × (NC*H*_2_)), 1.83 (quin, 4H, *J* = 6.6, Hz, 2 × CH_2_) ppm; ^13^C-NMR (151 MHz, DMSO-*d*_6_) δ_C_: 160.77 (C(O)), 148.76, 148.52 (C=N), 134.54, 127.56, 127.28, 126.44, 122.13, 55.16 (s, 4 × C*_piperazine_*), 52,85 (C-N*_amide_*), 45.29 (C-N*_piperazine_*), 25.30 ppm; Anal. Calcd. (%) for C_26_H_30_N_6_O_2_ × 2HCl: C, 58.76; H, 6.07; N, 15.81. Found (%): C 58.48; H 6.26; N, 15.71.

*3,3'-[(Butane-1,4-diylbis(oxy))bis(propane-3,1-diyl)]bis(quinazolin-4(3H)-one)* (**5b**). White solid. Yield 40%; M.p. 91.5–92.0 °C; ^1^H-NMR (600 MHz, DMSO-*d*_6_) δ_H_: 8.32 (s, 2H, N=C*H*), 8.16 (dd, 2H, *J* = 7.8, 1.2 Hz, *H_arom_*_._), 7.81 (ddd, 2H, *J* = 8.4, 7.8, 1.2 Hz, *H_arom_*_._), 7.67 (d, 2H, *J* = 8.4 Hz, *H_arom_*_._), 7.53 (ddd, 2H, *J* = 7.8, 7.8, 1.2 Hz, *H_arom_*_._), 4.04 (t, 4H, *J* = 7.0 Hz, 2 × NC*H*_2_), 3.40 (t, 4H, *J* = 6.0 Hz, 2 × N(CH_2_)_2_C*H*_2_O), 3.33–3.24 (m, 4H, 2 × OC*H*_2_), 1.94 (quin, 4H, *J* = 6.6 Hz, 2 × NCH_2_C*H*_2_), 1.41–1.39 (m, 4H, 2 × C*H_2_*CH_2_O) ppm; ^13^C-NMR (151 MHz, DMSO-*d*_6_) δ_C_: 160.74 (C(O)), 148.57, 148.43 (C=N), 134.58, 127.59, 127.34, 126.45, 122.07, 70.31 (O-C*_but_*_._), 67.74 (C*_prop_*_._-N), 44.51 (C-N*_amide_*), 28.86, 26.38 ppm; Anal. Calcd. (%) for C_23_H_30_N_4_O_4_: C, 67.51; H, 6.54; N, 12.11. Found (%): C, 67.43; H, 6.19; N, 12.09.

*3,3'-[(Methylazanediyl)bis(propane-3,1-diyl)]bis(quinazolin-4(3H)-one)* (**5c**). Colourless oil. Yield 37.4%; ^1^H-NMR (600 MHz, DMSO-*d*_6_) δ_H_: 8.38 (s, 2H, N=C*H*), 8.16 (dd, 2H, *J* = 7.9, 1.4 Hz, *H_arom_*_._), 7.82 (ddd, 2H, *J* = 8.4, 7.3, 1.2 Hz, *H_arom_*_._), 7.68 (d, 2H, *J* = 8.4 Hz, *H_arom_*_._), 7.55 (td, 2H, *J* = 7.9, 1.2 Hz, *H_arom_*_._), 4.04 (dd, 4H, *J* = 7.0, 6.9 Hz, 2 × NC*H*_2_), 3.32 (s, 3H, NC*H*_3_), 2.35 (t, 4H, *J* = 6.9 Hz, 2 × C*H*_2_NCH_3_), 1.83 (quin, 4H, *J* = 6.9 Hz, 2 × C*H_2_*CH_2_NCH_3_) ppm; ^13^C-NMR (151 MHz, DMSO-*d*_6_) δ_C_: 160.66 (C(O)), 148.61, 148.45 (C=N), 134.59, 127.59, 127.37, 126.46, 122.06, 54.63 (C-N), 45.07 (CH_3_), 41.60 (C-N*_amide_*), 26.49 ppm; Anal. Calcd. (%) for C_23_H_25_N_5_O_2_ × HCl × ^1^/_2_H_2_O: C, 61.53; H, 6.06; N, 15.60. Found (%): C, 61.40; H, 5.59; N, 15.67.

*3,3'-[Azanediylbis(propane-3,1-diyl)]bis(quinazolin-4(3H)-one)* (**5d**). White solid. Yield 69.8%; M.p. 94.0–95.8 °C; ^1^H-NMR (600 MHz, DMSO-*d*_6_) δ_H_: 8.37 (s, 2H, N=C*H*), 8.16 (dd, 2H, *J* = 8.0, 1.4 Hz, *H_arom_*_._), 7.82 (ddd, 2H, *J* = 8.3, 7.2, 1.4 Hz, *H_arom_*_._), 7.68 (d, 2H, *J* = 8.3 Hz, *H_arom_*_._), 7.55 (ddd, 2H, *J* = 8.0, 7.2, 1.1 Hz, *H_arom_*_._), 4.00 (t, 4H, *J* = 7.1 Hz, 2 × NC*H*_2_), 2.52 (brs, 1H, NH), 2.35 (t, 4H, *J* = 6.8 Hz, 2 × C*H*_2_NH), 1.85 (quin, 4H, *J* = 6.8 Hz, 2 × C*H_2_*CH_2_NH) ppm; ^13^C-NMR (151 MHz, DMSO-*d*_6_) δ_C_: 160.70 (C(O)), 148.60, 148.43 (C=N), 134.68, 127.59, 127.42, 126.47, 122.04, 46.55 (C-N), 44.83 (C-N*_amide_*), 29.23 ppm; Anal. Calcd. (%) for C_23_H_25_N_5_O_2_ × 3H_2_O: C, 59.58; H, 6.59; N, 15.79. Found (%): C, 59.95; H, 6.34; N, 15.58.

#### 3.2.4. General Procedure for the Synthesis of **6a**–**d**

A mixture of bisanthranilamide **3a**–**d** (10 mmol) with CDI (11 mmol) in DMF (100 mL) was heated at 70 °C for 5 h. After cooling, the crude product was filtered off, dried and crystallized from DMF/H_2_O.

*3,3'-[Piperazine-1,4-diylbis(propane-3,1-diyl)]bis(quinazoline-2,4-(1H,3H)-dione)* (**6a**). Creamy solid. Yield 70.80%; M.p. > 300 °C; ^1^H-NMR (300 MHz, DMSO-*d*_6_) δ_H_: 11.38 (brs, 1H, N*H*), 7.92 (dd, 2H, *J* = 7.9, 1.2 Hz, C*H_arom_*_._), 7.61 (ddd, *J* = 7.3, 7.3, 1.5 Hz, 2H, C*H_arom_*_._), 7.17 (m, 4H, C*H_arom_*_._), 3.91 (t, *J* = 6.8 Hz, 4H, 2 × NC*H*_2_), 2.40–2.04 (cluster, 12H, 4 × C*H*_2*piperazine*_ and 2 × C*H*_2_N), 1.69 (m, 4H, CH_2_C*H*_2_CH_2_) ppm; ^13^C-NMR (75 MHz, DMSO-*d*_6_) δ_C_: 161.68 (C(O)), 149.93 (C(O)), 139.18, 134.63, 127.16, 122.19, 114.85, 113.60, 55.10 (s, 4 × C*_piperazine_*), 52.80 (C-N*_amide_*), 45.28 (C-N*_piperazine_*), 25.29 ppm; Anal. Calcd. (%) for C_26_H_30_N_6_O_4_ × HCl × H_2_O: C 50.70; H 5.40; N 13.64. Found (%): C, 50.52; H, 5.80; N, 13.64.

*3,3'-[Butane-1,4-diylbis(oxypropane-3,1-diyl)]bis(quinazoline-2,4-(1H,3H)-dione)* (**6b**). Bright yellow solid. Yield 54.0%; M.p. 191.1–92.7 °C; ^1^H-NMR (300 MHz, DMSO-*d*_6_) δ_H_: 11.39 (s, 1H, N*H*), 7.92 (dd, 2H, *J* = 7.8, 1.2 Hz, C*H_arom_*_._), 7.64 (ddd, *J* = 7.4, 7.3, 1.5 Hz, 2H, C*H_arom_*_._), 7.18 (m, 4H, C*H_arom_*_._), 3.97 (t, 4H, *J* = 6.8 Hz, 2 × NC*H*_2_), 3.40 (dd, 4H, *J* = 6.2, 6.2 Hz, 2 × N(CH_2_)_2_C*H*_2_O), 3.29 (m, 4H, 2 × OC*H*_2_), 1.90–1.71 (m, 4H, 2 × NCH_2_C*H*_2_), 1.41–1.39 (m, 4H, 2 × C*H_2_*CH_2_O) ppm; ^13^C-NMR (75 MHz, DMSO-*d*_6_) δ_C_: 161.70 (C(O)), 149.95 (C(O)), 139.19, 134.63, 127.16, 122.20, 114.85, 113.66, 69.71 (O-C*_but_*_._), 68.09 (C*_prop_*_._-O), 37.99 (C-N*_amide_*), 27.67, 25.95 ppm; Anal. Calcd. (%) for C_26_H_30_N_4_O_6_: C, 63.14; H, 6.11; N, 11.33. Found (%):C, 62.89; H, 5.94; N, 11.40.

*3,3'-[(Methylazanediyl)bis(propane-3,1-diyl)]bis(quinazoline-2,4-(1H,3H)-dione)* (**6c**). White solid. Yield 63.6%; M.p. 223.6–225.3 °C; ^1^H-NMR (300 MHz, DMSO-*d*_6_) δ_H_: 11.40 (s, 1H, N*H*), 7.90 (dd, 2H, *J* = 7.8, 1.2 Hz, C*H_arom_*_._), 7.63 (ddd, *J* = 7.4, 7.3, 1.5 Hz, 2H, C*H_arom_*_._), 7.21–7.16 (m, 4H, C*H_arom_*_._), 3.95 (dd, 4H, *J* = 7.0, 6.9 Hz, 2 × NC*H*_2_), 2.35 (dd, 4H, *J* = 6.8, 6.8 Hz, 2 × C*H*_2_NCH_3_), 2.13 (s, 3H, NC*H*_3_), 1.75–1.60 (m, 4H, 2 × C*H_2_*CH_2_NCH_3_) ppm; ^13^C-NMR (75 MHz, DMSO-*d*_6_) δ_C_: 161.64 (C(O)), 149.90 (C(O)), 139.45, 134.62, 127.16, 122.20, 114.88, 113.65, 54.74 (C-N), 45.07 (CH_3_), 41.60 (C-N*_amide_*), 25.12 ppm; Anal. Calcd. (%) for C_23_H_25_N_5_O_4_ × HCl × 2^1^/_2_H_2_O: C, 53.43; H 6.04; N, 13.55. Found (%): C, 53.06; H, 5.91; N, 13.53.

*3,3'-[Azanediylbis(propane-3,1-diyl)]bis (quinazoline-2,4-(1H,3H)-dione)* (**6d**). White solid. Yield 83%; M.p. 210.1–211.2 °C; ^1^H-NMR (300 MHz, DMSO-*d*_6_) δ_H_: 11.39 (s, 1H, N*H*), 7.90 (dd, 2H, *J* = 7.8, 1.2 Hz, C*H_arom_*_._), 7.64 (ddd, *J* = 7.4, 7.3, 1.5 Hz, 2H, C*H_arom_*_._), 7.20–7.15 (m, 4H, C*H_arom_*_._), 3.95 (dd, 4H, *J* = 7.0, 6.9 Hz, 2 × NC*H*_2_), 2.35 (dd, 4H, *J* = 6.8, 6.8 Hz, 2 × C*H*_2_N), 1.76–1.59 (m, 4H, 2 × C*H*_2_CH_2_N) ppm; ^13^C-NMR (75 MHz, DMSO-*d*_6_) δ: 161.62 (C(O)), 149.91 (C(O)), 139.48, 134.63, 127.15, 122.21, 114.89, 113.62, 54.73 (C-N), 41.61 (C-N*_amide_*), 25.11 ppm; Anal. Calcd. for C_22_H_23_N_5_O_4_: C, 62.70; H, 5.50; N, 16.62. Found (%): C, 62.91; H, 5.40; N, 16.22.

#### 3.2.5. General Procedure for the Synthesis of **11a**–**d** to **14a**–**d**

A mixture of acid **10**–**13** (10 mmol) and CDI (10 mmol) in acetonitrile (100 mL) or DMF (100 mL) was stirred for 1 h at room temperature. Then the appropriate polyamine (**a**–**d**) (6 mmol) was added and stirring was continued for additional 2 h, then the mixture was filtered. The solvent was removed under reduced pressure and 20 mL of H_2_O was added to the residue and left for 24 h at 5 °C (for compounds **11a**–**d**, **13a**–**d**, **14a**–**d**. Then the solid was filtered off, washed with H_2_O and crystallized from DMF/H_2_O. In case of compounds **12a**–**d** the residues were purified by column chromatography over silica gel (CHCl_3_/MeOH, 100:1, 10:1, *v*/*v*).

*N,N'-[Piperazine-1,4-diylbis(propane-3,1-diyl)]bis(2-naphthamide)* (**11a**). White solid. Yield 52.1%; M.p. 164.8–66.2 °C; ^1^H-NMR (600 MHz, DMSO-*d*_6_) δ_H_: 8.64 (t, 2H, *J* = 5.3 Hz, 2 × CON*H*), 8.43 (s, 2H, C*H_arom._*), 8.05–7.91 (cluster, 8H, C*H_arom._*), 7.62–7.56 (cluster, 4H, C*H_arom._*), 3.36 (ddd, 4H, *J* = 7.0, 7.0, 5.3 Hz, C*H*_2_N), 2.48–2.31 (cluster, 12H, 4 × C*H*_2*piperazine*_ and 2 × NC*H*_2_), 1.73 (quin, 4H, *J* = 7.0 Hz, CH_2_C*H*_2_CH_2_) ppm; ^13^C-NMR (151 MHz, CDCl_3_) δ_C_: 167.42 (C(O)), 134.72, 132.64, 132.20, 128.81, 128.26, 127.79, 127.49, 127.32, 126.66, 123.96, 58.33 (C-N*_piperazine_*), 53.46(s, 4 × C*_piperazine_*), 40.86 (C-N*_amide_*), 24.53 ppm; Anal. Calcd. (%) for C_32_H_36_N_4_O_2_ × 2HCl × 2H_2_O: C, 62.23; H, 6.85; N, 9.07. Found (%): C, 62.62; H, 6.79; N, 9.26.

*N,N'-[(Butane-1,4-diylbis(oxy)bis(propane-3,1-diyl)]bis(2-naphthamide)* (**11b**). Yellow solid. Yield 42.7%; M.p. 154.9–55.3 °C; ^1^H-NMR (600 MHz, DMSO-*d*_6_) δ_H_: 8.58 (t, 2H, *J* = 5.4 Hz, 2 × CON*H*), 8.44 (s, 2H, C*H_arom._*), 8.03–7.93 (cluster, 8H, C*H_arom._*), 7.63–7.58 (m, 4H, C*H_arom._*), 3.46 (dd, 4H, *J* = 6.6, 5.4 Hz, C*H*_2_N), 3.41–3.37 (cluster, 8H, 2 × C*H*_2_O and 2 × NC*H*_2_), 1.81 (quin, 4H, *J* = 6.6 Hz, 2 × NCH_2_C*H*_2_), 1.60–1.54 (m, 4H, 2 × C*H_2_*CH_2_O) ppm; ^13^C-NMR (151 MHz, CDCl_3_) δ_C_: 167.42 (C(O)), 134.72, 132.64, 132.20, 128.81, 128.26, 127.79, 127.49, 127.32, 126.66, 123.96, 69.33 (O-C*_but_*_._), 65,89 (C*_prop_*_._-O), 33,46 (C-N*_amide_*), 30.86, 25.53 ppm; Anal. Calcd. for C_32_H_36_N_4_O_2_: C, 74.97, H, 7.08, N, 5.46. Found (%): C, 74.55, H, 7.21, N, 5.65.

*N,N'-[(Methylazanediyl)bis(propane-3,1-diyl)]bis(2-naphthamide)* (**11c**). White solid. Yield 54.6%; M.p. 136.8–138.0 °C; ^1^H-NMR (600 MHz, CDCl_3_) δ_H_: 8.67 (s, 2H, 2 × CON*H*), 7.82 (s, 2H, C*H_arom._*), 8.03–7.92 (cluster, 8H, C*H_arom._*), 7.61–7.59 (cluster, 4H, C*H_arom._*), 3.60 (q, 4H, *J* = 6.4, 2 × NC*H*_2_), 2.56 (dd, 4H, *J* = 6.4, 6.4 Hz, 2 × C*H*_2_NCH_3_), 2.34 (s, 3H, NC*H*_3_), 1.85 (quin, 4H, *J* = 6.4 Hz, 2 × CH_2_C*H*_2_CH_2_) ppm; ^13^C-NMR (151 MHz, CDCl_3_) δ_C_: 167.48 (C(O)), 134.58, 132.59, 131.97, 128.84, 128.22, 127.63, 127.41, 127.35, 126.56, 123.66, 56.41 (C-N), 41.88 (CH_3_), 39.27 (C-N*_amide_*), 26.61 ppm; Anal. Calcd. (%) for C_29_H_31_N_3_O_2_: C, 76.62; H, 7.09; N, 9.24. Found (%): C, 76.35; H, 6.85; N, 9.52.

*N,N'-[Azanediylbis(propane-3,1-diyl)]bis(2-naphthamide)* (**11d**). White solid. Yield 55.2%; M.p. 158.5–160.0 °C; ^1^H-NMR (600 MHz, DMSO-*d*_6_) δ_H_: 8.66 (t, 2H, *J* = 5.4 Hz, 2 × CON*H*), 8.43 (s, 2H, C*H_arom._*), 8.03–7.93 (cluster, 8H, C*H_arom._*), 7.61–7.54 (cluster, 4H, C*H_arom._*), 3.38–3.26 (m, 4H, NC*H*_2_), 2.47–2.34 (cluster, 5H, N*H*, 2 × C*H*_2_NCH_3_), 1.73 (quin, 4H, *J* = 6.5 Hz, 2 × CH_2_C*H*_2_CH_2_) ppm; ^13^C-NMR (151 MHz, DMSO-*d*_6_) δ_C_: 166.68 (C(O)), 134.54, 132.66, 132.56, 129.25, 128.34, 128.28, 128.06, 127.72, 127.12, 127.12, 124.64, 47.56 (C-N), 39.63 (C-N), 38.33 ppm; Anal. Calcd. (%) for C_28_H_29_N_3_O_2_ × H_2_O: C, 73.34; H, 7.03; N, 9.16. Found (%): C, 73.46, H, 6.74, N, 9.40.

*N,N'-[Piperazine-1,4-diylbis(propane-3,1-diyl)]bis(quinoline-2-carboxamide)* (**12a**). White needles. Yield 49.8%; M.p. 205.2–205.7 °C; ^1^H-NMR (600 MHz, CDCl_3_) δ_H_: 8.77 (t, 2H, *J* = 5.6 Hz, 2 × CON*H*), 8.33 (dd, 4H, *J* = 8.5, 5.5 Hz, C*H_arom_*_._), 8.15 (d, 2H, *J* = 8.5 Hz, C*H_arom_*_._), 7.89 (d, 2H, *J* = 8.5 Hz, C*H_arom_*_._), 7.73 (dd, 2H, *J* = 7.5, 7.5 Hz, C*H_arom_*_._), 7.59 (dd, 2H, *J* = 7.5, 7.5 Hz, C*H_arom._*), 3.65 (q, 4H, *J* = 6.5 Hz, 2 × NC*H*_2_), 3.46–2.72 (cluster, 12H, 4 × C*H*_2*piperazine*_ and 2 × C*H*_2_N), 1.89 (quin, 4H, *J* = 6.5 Hz, 2 × CH_2_C*H*_2_CH_2_) ppm; ^13^C-NMR (151 MHz, CDCl_3_) δ_C_: 164.50 (C(O)), 150.16 (C=N), 146.45, 137.26, 129.82, 129.65, 129.20, 127.67, 118.96, 57.02 (s, 4 × C*_piperazine_*), 53,31 (C-N*_piperazine_*), 38.87 (C-N*_amide_*), 26.26 ppm; Anal. Calcd. (%) for C_30_H_34_N_6_O_2_: C, 70.56; H, 6.71; N, 16.46. Found (%): C, 70.32; H, 6.80; N, 16.85.

*N,N'-[(Butane-1,4-diylbis(oxy))bis(propane-3,1-diyl)]bis(quinoline-2-carboxamide)* (**12b**). White needles. Yield 46.50%; M.p. 103.5–104.6 °C; ^1^H-NMR (600 MHz, DMSO-*d*_6_) δ_H_: 8.89 (t, 2H, *J* = 5.7 Hz, 2 × CON*H*), 8.55 (d, 2H, *J* = 8.5 Hz, C*H_arom_*_._), 8.15 (d, 2H, *J* = 8.5 Hz, C*H_arom_*_._), 8.12 (d, 2H, *J* = 8.5 Hz, C*H_arom_*_._), 8.07 (d, 2H, *J* = 8.0 Hz, C*H_arom_*_._), 7.86 (dd, 2H, *J* = 8.5, 8.0 Hz, C*H_arom_*_._), 7.70 (dd, 2H, *J* = 8.0, 8.0 Hz, C*H_arom_*_._), 3.50–3.25 (cluster, 12H, 2 × NC*H*_2_ and 2 × N(CH_2_)_2_C*H*_2_O and 2 × OC*H*_2_), 1.83 (quin, 4H, *J* = 6.5, 6.5, 6.5, 6.5 Hz, 2 × NCH_2_C*H*_2_), 1.61–1.59 (m, 4H, 2 × C*H_2_*CH_2_O)ppm. ^13^C-NMR (151 MHz, CDCl_3_) δ_C_: 164.51 (C(O)), 150.14 (C=N), 146.43, 137.28, 129.81, 129.64, 129.22, 127.68, 118.98, 69.75 (O-C*_but_*_.)_, 68.02 (C*_prop_*_._-O), 37.96 (C-N*_amide_*), 27.68, 25.93 ppm; Anal. Calcd. (%) for C_30_H_34_N_4_O_4_: C, 70.02; H, 6.66; N, 10.89. Found (%): C, 70.02; H, 6.74; N, 10.98.

*N,N'-[(Methylazanediyl)bis(propane-3,1-diyl)]bis(quinoline-2-carboxamide)* (**12c**). Yellow oil. Yield 52.9%; ^1^H-NMR (600 MHz, DMSO-*d*_6_) δ_H_: 9.10 (t, 2H, *J* = 5.6 Hz, 2 × CONH), 8.50 (d, 4H, *J* = 8.4 Hz, C*H_arom_*_._), 8.13 (d, 2H, *J* = 8.4 Hz, C*H_arom_*_._), 8.10 (d, 2H, *J* = 8.4 Hz, C*H_arom_*_._), 8.05 (d, 2H, *J* = 8.2 Hz, C*H_arom_*_._), 7.80 (dd, 2H, *J* = 8.1, 7.2 Hz, C*H_arom_*_._), 7.67 (dd, 2H, *J* = 7.2, 7.2 Hz, C*H_arom_*_._), 3.47 (q, 4H, *J* = 6.5 Hz, NC*H*_2_), 2.37 (dd, 4H, *J* = 6.5, 6.5 Hz, C*H*_2_NCH_3_), 2.25 (s, 3H, NC*H*_3_), 1.81 (quin, 4H, *J* = 6.5 Hz, CH_2_C*H*_2_CH_2_) ppm; ^13^C-NMR (150 MHz, DMSO-*d*_6_) δ_C_: 164.29 (C(O)), 150.77 (C=N), 146.47, 137.18, 130.81, 129.60, 129.20, 128.48, 128.34, 119.04, 56.02 (C-N), 42.19 (CH_3_), 38.45 (C-N*_amide_*), 27.16 ppm; Anal. Calcd. (%) for C_27_H_29_N_5_O_2_ × 2^1^/_2_H_2_O: C, 64.78, H, 6.84, N, 13.99. Found (%): C, 64.77, H, 6.14, N, 13.59.

*N,N'-[Azanediylbis(propane-3,1-diyl)]bis(quinoline-2-carboxamide)* (**12d**). Yellow oil. Yield 50.6%; M.p. 186.4–88.0 °C; ^1^H-NMR (600 MHz, CDCl_3_) δ_H_: 8.73 (t, 2H, *J* = 6.4 Hz, 2 × CON*H*), 8.09 (d, 2H, *J* = 8.5 Hz, C*H_arom_*_._), 7.83 (d, 2H, *J* = 8.0 Hz, C*H_arom_*_._), 7.73 (td, 2H, *J* = 7.0, 1.32 Hz, C*H_arom_*_._), 7.60 (dd, 2H, *J* = 7.0, 7.0 Hz, C*H_arom_*_._), 3.77 (q, 4H, *J* = 6.5 Hz, 2 × NC*H*_2_), 3.20–3.10 (m, 4H, C*H*_2_NH), 2.39 (quin, 4H, *J* = 6.5, 6.5, 6.5, 6.5 Hz, CH_2_C*H*_2_CH_2_), 2.30 (brs, 1H, N*H*), ppm; ^13^C-NMR (150 MHz, CDCl_3_) δ_C_: 165.89 (C(O)), 148.97 (C=N), 146.48, 137.50, 130.20, 129.79, 129.32, 128.07, 127.64, 118.65, 45.74 (C-N), 36.45 (C-N*_amide_*), 26.73 ppm; Anal. Calcd. (%) for C_26_H_27_N_5_O_2_ × HCl × H_2_O: C, 62.96; H, 6.10; N, 14.12. Found (%): C, 63.35; H, 5.81; N, 14.48.

*N,N'-[Piperazine-1,4-diylbis(propane-3,1-diyl)]bis(2-oxochromane-3-carboxamide)* (**13a**). Creamy solid. Yield 77.9%; M.p. 201.0–201.7 °C; ^1^H-NMR (600, MHz, CDCl_3_) δ_H_: 8.93 (t, 2H, *J* = 5.4 Hz, 2 × CON*H*), 8.92 (s, 2H, C*H_arom_*_._), 7.71 (dd, 4H, *J* = 7.8, 1.2 Hz, C*H_arom_*_._), 7.68 (ddd, 4H, *J* = 8.4, 7.8, 1.2 Hz, C*H_arom_*_._), 7.42 (d, 4H, *J* = 8.4 Hz, C*H_arom_*) 7.30 (td, 4H, *J* = 7.8, 1.2 Hz, C*H_arom_*), 3.55 (q, 4H, *J* = 6.8 Hz, 2 × C*H*_2_N), 2.65–2.40 (cluster, 12H, 4 × C*H*_2*piperazine*_ and 2 × NC*H*_2_), 1.84 (quin, 4H, *J* = 7.0 Hz, 2 × CH_2_C*H*_2_CH_2_) ppm; ^13^C-NMR (151 MHz, CDCl_3_) δ_C_: 161.42 (C(O)), 161.27 (C(O)), 154.44, 148.10, 133.87, 129.73, 125.20, 118.71, 116.60, 56.06 (s, 4 × C*_piperazine_*), 53,21 (C-N*_piperazine_*), 38.35 (C-N*_amide_*), 26.46 ppm; Anal. Calcd. (%) for C_30_H_34_N_4_O_6_ × 2HCl × 2H_2_O: C, 54.96; H, 6.15; N, 8.55. Found (%): C, 54.60; H, 5.79; N, 8.89.

*N,N'-[(Butane-1,4-diylbis(oxy))bis(propane-3,1-diyl)]bis(2-oxo-2H-chromene-3-carboxamide)* (**13b**). Yellow needles. Yield 59%; M.p. 101.2–102.0 °C; ^1^H-NMR (DMSO-*d*_6_) δ_H_: 8.96 (s, 2H, 2 × CON*H*), 8.91 (s, 2H, C*H_arom_*), 7.70 (dd, *J* = 7.7, 1.5 Hz, 2H, C*H_arom_*_._), 7.67 (ddd, *J* = 8.4, 7.7, 1.5 Hz, 2H, C*H_arom_*_._), 7.42 (d, *J* = 8.4 Hz, 2H, C*H_arom_*_._), 7.39 (td, *J* = 7.7, 1.5 Hz, 2H, C*H_arom_*_._), 3.60–3.51 (cluster, 12H, 2 × NC*H*_2_ and 2 × N(CH_2_)_2_C*H*_2_O and 2 × OC*H*_2_), 1.92 (quin, 4H, *J* = 6.5 Hz, 2 × NCH_2_C*H*_2_), 1.6–1.59 (m, 4H, 2 × C*H_2_*CH_2_O) ppm; ^13^C-NMR (151 MHz, CDCl_3_) δ_C_: 161.50 C(O)), 161.30 (C(O)), 154.45, 148.11, 133.90, 129.80, 125.25, 118.71, 116.62, 69.74 (O-C*_but_*_._), 68.00 (C*_prop_*_._-O), 37.95 (C-N*_amide_*), 27.70, 25.90 ppm; Anal. Calcd. (%) for C_30_H_32_N_2_O_8_ × H_2_O: C, 63.59; H, 6.05; N, 4.94. Found (%): C, 63.92; H, 6.05; N, 5.25.

*N,N'-[(Methylazanediyl)bis(propane-3,1-diyl)]bis(2-oxo-2H-chromene-3-carboxamide)* (**13c**). White solid. Yield 56.8%; M.p. 263.5–264.5 °C; ^1^H-NMR (600 MHz, CDCl_3_) δ_H_: 9.01 (brs, 2H, 2 × CON*H*), 8.89 (s, 2H, C*H_arom_*), 7.69 (dd, 2H, *J* = 7.7, 1.3 Hz, C*H_arom_*), 7.65 (ddd, 2H, *J* = 8.6, 7.2, 1.5 Hz, C*H_arom_*), 7.41–7.35 (m, 4H, C*H_arom_*),3.57 (q, 4H, *J* = 6.6 Hz, 2 × NC*H*_2_), 2.51 (dd, 4H, *J* = 6.9, 6.9 Hz, 2 × C*H*_2_NH), 2.28 (s, 3H, NC*H*_3_), 1.84 (quin, 4H, *J* = 6.9 Hz, 2 × CH_2_C*H*_2_CH_2_) ppm; ^13^C-NMR (151 MHz, CDCl_3_) δ_C_: 161.99 (C(O)), 160.68 (C(O)), 154.33, 147.82, 134.57, 130.70, 125.62, 119.54, 118.87, 116.59, 53.18 (C-N), 49.66 (CH_3_), 37.00 (C-N*_amide_*), 24.18 ppm; Anal. Calcd. (%) for C_27_H_29_N_3_O_6_ × HCl × 1^1^/_2_H_2_O: C, 58.43, H, 5.99, N, 7.57. Found (%): C, 57.98, H, 5.59, N, 7.59.

*N,N'-[Azanediylbis(propane-3,1-diyl)]bis(2-oxo-2H-chromene-3-carboxamide)* (**13d**). Creamy solid. Yield: 62.1%; M.p. 189.0–191.0 °C; ^1^H-NMR (600 MHz, DMSO-*d*_6_) δ_H_: 8.83 (s, 2H, C*H_arom_*), 8.77 (t, 2H, *J* = 5.6 Hz, 2 × CON*H*), 7.96 (dd, 2H, *J* = 7.8, 1.4 Hz, C*H_arom_*), 7.73 (ddd, 2H, *J* = 8.1, 7.6, 1.6 Hz, C*H_arom_*), 7.48 (d, 2H, *J* = 8.1 Hz, C*H_arom_*), 7.43 (td, 2H, *J* = 7.6, 7.6, 0.6 Hz, C*H_arom_*), 3.41 (q, 4H, *J* = 6.8 Hz, 2 × NC*H*_2_), 2.59 (t, 4H, *J* = 6.7 Hz, 2 × C*H*_2_NH), 1.76–1.62 (brs, 1H, N*H*), 1.69 (quin, 4H, *J* = 6.7 Hz, 2 × CH_2_C*H*_2_CH_2_) ppm**;** ^13^C-NMR (151 MHz, DMSO-*d*_6_) δ_C_: 162.12 (C(O)), 161.63 (C(O)), 154.33, 147.86, 134.61, 130.72, 125.64, 119.50, 118.87, 116.60, 45.20 (C-N), 36.84 (C-N*_amide_*), 26.32 ppm; Anal. Calcd. (%) for C_26_H_27_N_3_O_6_ × HCl × 1^1^/_2_H_2_O: C, 53.34, H, 5.34, N, 7.18. Found (%): C, 53.38, H, 4.97, N, 7.23.

*N,N'-[Piperazine-1,4-diylbis(propane-3,1-diyl)]bis(1H-indole-2-carboxamide)* (**14a**). Creamy solid. Yield 52.9%; M.p. 246.0 °C with decomp.; ^1^H-NMR (600 MHz, DMSO-*d*_6_) δ_H_: 11.51 (brs, 2H, N*H_indole_*), 8.45 (t, 2H, *J* = 5.5 Hz, 2 × CON*H*), 7.77 (d, 2H, *J* = 8.0, C*H_arom_*_._), 7.43 (dd, 2H, *J* = 8.1, 1.0 Hz, C*H_arom_*_._), 7.14 (dt, 2H, *J* = 8.2, 7.0, 1.0 Hz, CH*_arom_*_._), 7.43 (s, C*H_indole_*), 7.02 (dt, 2H, *J* = 8.0, 7.0, 0.6 Hz, C*H_arom_*_._), 3.34–3.27 (m, 4H, C*H*_2_N), 2.20–2.47 (cluster, 12H, 2 × C*H*_2*piperazine*_, 2 × NC*H*_2_), 1.68 (quin, 4H, *J* = 6.9 Hz, 2 × CH_2_C*H*_2_CH_2_) ppm; ^13^C-NMR (151 MHz, DMSO-*d*_6_) δ_C_: 161.81 (C(O)), 136.92, 132.06, 127.52, 123.78, 121.92, 120.20, 112.77, 103.23, 54.41 (s, 4 × C*_piperazine_*), 48.72 (C-N*_piperazine_*_)_, 36.48 (C-N*_amide_*_)_, 24,30 ppm; Anal. Calcd. (%) for C_28_H_34_N_6_O_2_ × 2HBr × 1^1^/_2_H_2_O: C, 52.26, H, 6.11, N, 13.06. Found (%): C, 52.23, H, 6.38, N, 13.06.

*N,N'-[(Butane-1,4-diylbis(oxy))bis(propane-3,1-diyl)]bis(1H-indole-2-carboxamide)* (**14b**). Creamy solid. Yield 43.9%; M.p. 182.3–183.1 °C; ^1^H-NMR (600 MHz, DMSO-*d*_6_) δ_H_: 11.52 (brs, 2H, N*H_indole_*), 8.41 (t, 2H, *J* = 6.0 Hz, 2 × CON*H*), 7.61 (d, 2H, *J* = 8.4 Hz, C*H_arom_*_._), 7.43 (d, 2H, *J* = 7.8 Hz, C*H_arom_*_._), 7.17 (dt, 2H, *J* = 8.4, 7.0, 1.2 Hz, C*H_arom_*_._), 7.10 (s, C*H_indole_*), 7.03 (dt, 2H, *J* = 7.8, 7.0, 0.8 Hz, C*H_arom_*_._), 3.44 (t, 4H, *J* = 6.6, 6.0 Hz, C*H*_2_N), 3.40–3.34 (cluster, 8H, 2 × C*H*_2_O and 2 × NC*H*_2_), 1.79 (quin, 4H, *J* = 6.6 Hz, 2 × NCH_2_C*H*_2_), 1.57–1.55 (m, 4H, 2 × C*H_2_*CH_2_O) ppm; ^13^C-NMR (151 MHz, DMSO-*d*_6_) δ_C_: 161.81 (C(O)), 136.92, 132.06, 127.52, 123.78, 121.92, 120.20, 112.77, 103.23, 69.71 (O-C*_but_*_._), 68.09 (C*_prop_*_._-O), 37. 99 (C-N*_amide_*), 27.67, 25.95 ppm; Anal. Calcd. for C_28_H_34_N_4_O_4_ × HCl × ^1^/_2_H_2_O, (%): C, 62.94, H, 7.04, N, 15.29. Found (%): C, 62.93, H, 6.70, N, 15.30.

*N,N'-[(Methylazanediyl)bis(propane-3,1-diyl)]bis(1H-indole-2-carboxamide)* (**14c**). Creamy solid. Yield 52.0%; M.p. 205.3–207.0 °C; ^1^H-NMR (600 MHz, DMSO-*d*_6_) δ_H_: 11.52 (brs, 2H, N*H_indole_*), 8.50 (t, 2H, *J* = 5.4 Hz, 2 × CON*H*), 7.60 (d, 2H, *J* = 7.8 Hz, C*H_arom_*_._), 7.44 (d, 2H, *J* = 8.4 Hz, C*H_arom_*_._), 7.17 (dd, 2H, *J* = 8.4, 7.2 Hz, C*H_arom_*_._), 7.09 (s, C*H_indol_*_e_), 7.03 (dd, 2H, *J* = 7.8, 7.2 Hz, C*H_arom_*_._), 3.36–3.33 (m, 4H, 2 × NC*H*_2_), 2.40 (dd, 4H, *J* = 7.2, 7.2 Hz, 2 × C*H*_2_NCH_3_), 2.19 (s, 3H, NC*H*_3_), 1.85 (quin, 4H, *J* = 7.2 Hz, 2 × CH_2_C*H*_2_CH_2_) ppm; ^13^C-NMR (151 MHz, DMSO-*d*_6_) δ_C_: 161.81 (C(O)), 136.92, 132.06, 127.52, 123.78, 121.92, 120.20, 112.77, 103.23, 56.02 (C-N), 42.19 (CH_3_), 38.45 (C-N*_amide_*), 27.16 ppm; Anal. Calcd. (%) for C_25_H_29_N_5_O_2_ × ^1^/_2_H_2_O: C, 68.16; H, 6.86; N, 15.90. Found (%): C, 67.94; H, 6.88; N, 16.00.

*N,N'-[Azanediylbis(propane-3,1-diyl)]bis(1H-indole-2-carboxamide)* (**14d**). Creamy solid. Yield 42.8%; M.p. 184.3–186.1 °C; ^1^H-NMR (600 MHz, DMSO-*d*_6_) δ_H_: 11.52 (brs, 2H, N*H_indol_*_e_), 8.50 (t, 2H, *J* = 5.4 Hz, 2 × CON*H*), 7.60 (d, 2H, *J* = 7.8 Hz, C*H_arom_*_._), 7.44 (d, 2H, *J* = 8.4 Hz, C*H_arom_*_._), 7.17 (dd, 2H, *J* = 8.4, 7.8 Hz, C*H_arom_*_._), 7.09 (s, 2H, C*H_indol_*_e_), 7.03 (dd, 2H, *J* = 7.8, 7.8 Hz, C*H_arom_*_._), 3.39–3.28 (m, 4H, 2 × NCH_2_, NH), 2.61 (t, 4H, *J* = 7.0 Hz, 2 × C*H*_2_NH), 1.71 (quin, 4H, *J* = 7.0 Hz, 2 × CH_2_CH_2_CH_2_) ppm; ^13^C-NMR (151 MHz, DMSO-*d*_6_) δ_C_: 161.81 (C(O)), 136.93, 132.11, 127.53, 123.76, 121.93, 120.19, 112.77, 103.28, 45.36 (C-N), 36.43 (C-N*_amide_*), 26.56 ppm; Anal. Calcd. (%) for C_24_H_27_N_5_O_2_ × HCl: C, 62.94; H, 7.04; N, 15.29. Found (%): C, 62.93; H, 6.80; N, 15.30.

### 3.3. Biological In Vitro Evaluation

All chemicals used in bioassay were purchased from Sigma-Aldrich (Poznan, Poland), unless otherwise indicated. Roswell Park Memorial Institute 1640 Medium (RPMI), Dulbecco's Modified Eagle Medium (DMEM) and Foetal Bovine Serum (FBS) were purchased from Thermo Fisher Scientific Inc/Life Technologies (Warsaw, Poland).

#### 3.3.1. Preparation of Drug Stock and Working Solutions

Compounds **4a**, **12d** and **14d** were dissolved in sterile deionized water at a concentration of 1000, 2000 and 1000 µM, respectively, to prepare the corresponding stock solutions. Compounds **11c**, **11d** and **14c** were dissolved in dimethyl sulfoxide (DMSO) at a concentration of 50,000 µM (stock solution). Stock solutions were diluted to various concentrations with serum-free culture medium (RPMI or DMEM). Working solutions were used immediately to the experimental procedure. The final concentration of DMSO did not exceed an amount that had any detectable effect on cell growth.

#### 3.3.2. Cell Culture

Cell lines were initially purchased from American Type Culture Collection (ATCC™, Manassas, VA, USA) or European Collection of Cell Culture (ECACC^®^, Salisbury, UK). The PC–3 (CRL–1435™) prostate adenocarcinoma cells, the MCF–7 (86012803) mammary gland adenocarcinoma were routinely cultured in RPMI–1640 medium supplemented with 10% FBS. The DU–145 (HTB–81™) prostate carcinoma cells were routinely cultured in DMEM supplemented with 10% FBS. Cultures were maintained in 5% carbon dioxide at a temperature of 37 °C. Before each experiment, cells were serum deprived for 24 h. After pre-incubation, the cell culture media were replaced with drug-containing media. The cells were exposed to drugs for 48 h, followed by cell viability assessment for single drug treatments as described below.

#### 3.3.3. Drug Treatment

Half maximal inhibitory concentration (IC_50_) values of all examined compounds were determined for each cell line. Drug concentrations ranged from 5 to 50 µM for the single-drug treatment.

#### 3.3.4. WST–1 Cell Viability Assay

PC–3, DU–145, MCF–7 cells were plated in 96-well plates at a density of 1 × 10^4^ well in 100 μL of culture medium*.* Cell viability was estimated on the base of mitochondrial metabolic activity using the WST–1 assay (Roche, Basel, Switzerland) as describe elsewhere [37]. Ten µL of the WST–1 cell reagent was added to each well, after mixing gently to ensure homogeneous distribution of colour, the cells were incubated for additional 4 h at 37 °C. The absorbance of each well was measured using a microplate-reader (ELX808IU, BioTek, Winooski, VT, USA) at a wavelength of 450 nm. Relative cell viability (%) was expressed as a percentage relative to the untreated control cells. GraphPad Prism (version 5.01 for Windows, GraphPad Software Inc., San Diego, CA, USA) was employed to produce dose-response curves by performing nonlinear regression analysis. The viability of the treated cells was normalized to the viability of the untreated (control) cells, and cell viability fractions were plotted versus drug concentrations in the logarithmic scale. IC_50_ values were reported as mean values [47,48]. All experiments were performed in triplicates.

### 3.4. DNA Interaction Studies

#### 3.4.1. Thermal Melting Studies

In this study the following 29-mer oligonucleotides: 5’-AAA TTA ATA TGT ATT GTA TAT AAA TTA TT-3’ and 3’-TTT AAT TAT ACA TAA CAT ATA TTT AAT AA-5’ were employed. Oligonucleotides were purchased as HPLC-purified compounds from the Bioorganic Chemistry Department, Polish Academy of Science (Lodz, Poland; Geneworld synthesizer, K&A Laborgeraete GbR, Schaafheim, Germany) using nucleotide phosphoroamidites synthons as substrates (ChemGenes Corporation, Wilmington, MA, USA). The hybridization was carried out in reaction volume of 1 mL containing: single stranded oligonucleotides, 0.1 M NaCl, 0.01 M MgCl_2_, by heating to 90 °C for 10 min followed by slow cooling to room temperature in the presence or absence of different drug concentrations. The following compounds were employed: **11c**, **11d** (15 μM), DMSO control (final concentration was 0.1%) and 9-Aminoacridine hydrochloride hydrate **9AA** (Sigma-Aldrich, Saint Louis, MO, USA) (100 μM) as a positive control. DNA melting points were determined spectrophotometrically on a Cary 1.3E UV–Vis spectrophotometer (Agilent Technologies, Santa Clara, CA, USA) using a computer equipped with Cary WinUV software (Agilent Technologies). The absorbance changes at 260 nm was measured every minute in the range of 21–80 °C with an increment of 1 °C/min and 1 min as equilibration time. *T*_m_ values were obtained from the midpoint of the first-derivative plots. Experiments were performed in triplicate [38].

#### 3.4.2. Strains and Media

*Escherichia coli* DH5α cells with the plasmid pENTR4 were supplied from the Pharmaceutical Biotechnology Department, Medical University of Lodz (Lodz, Poland). Luria Broth (LB) medium (10 g tryptone, 5 g yeast extract, 2 g glucose and 10 g NaCl per liter of medium) was used for the growth of all cultures.

#### 3.4.3. Bacterial Culture and Plasmid Isolation

Agar plate supplemented with kanamycin (30 μg/mL) was inoculated with *E. coli* DH5α containing pENTR4 plasmid and incubated overnight, at 37 °C. The bacterial colonies were resuspended and subsequently, 250 mL of LB medium supplemented with kanamycin (30 μg/mL) was inoculated with the overnight culture equivalent to the 0.5 McFarland. The culture was incubated for 13 h at 37 °C with vigorous shaking (150 rpm). Plasmid was isolated from bacteria using a Plasmid Mini DNA purification system (A&A Biotechnology, Gdynia, Poland) as described by the manufacturer. Then supercoiled form was isolated from agarose gel using a Gel-Out Kit (A&A Biotechnology) as described by the manufacturer.

#### 3.4.4. Topoisomerase I Activity Assay

Topoisomerase I activity assay was carried out according to the method described by Sappal et al. [49] with a few modifications. Supercoiled pENTR4 DNA (0.2 μg) was a substrate for the reaction. Plasmid was incubated with 2 units of topoisomerase I in reaction volume of 20 mL (10 mM Tris-HCl, pH 7.5), 175 mM KCl, 5 mM MgCl_2_, 0.1 mM EDTA and 2.5% glycerol) in the presence of varying concentrations of the drug under study: **11c** (1–30 μM) and **11d** (1–30 μM), DMSO control (final concentration was 0.1% in all samples) and **9AA** (100 μM) as a positive control. Reactions were started after addition of the enzyme and stopped after 60 min at 37 °C by extracting the plasmid DNA with phenol–chloroform (*v*/*v*) following by adding stop solution (0.77% SDS, 0.77 mM EDTA, pH 8.0). Samples were then added to electrophoresis dye mixture (Polgen, Lodz, Poland), loaded onto 1% agarose gel running 1.5–2 V/cm in TAE buffer (40 mM Tris-acetate, pH 8.5, and 10 mM EDTA). The gels were stained with ethidium bromide 0.5 μg/mL, observed at UV light (260 nm) and photographed using a Gel Doc system (Syngene, Cambridge, UK).

### 3.5. Preliminary in Silico ADME Screening

All the predictions were performed using ACD/Percepta software obtained from ACD/Labs Inc. Toronto, ON, Canada [41]. *Drug-likeness* was evaluated using Drug Profiler Module. The default values for *drug-likeness* were set according to the published article [42]. Human Intestinal Absorption module allowed a quantitative estimation of maximum intestinal passive absorption of a compound (expressed as a percentage value and denoted %HIA) and the relative contributions from the transcellular and paracellular routes of absorption, calculated as a function of compound structure, lipophilicity and ionization constants, estimated human jejunal permeability coefficients at pH 6.5 (*P_e_*, 10^−4^ cm/s) and calculated intestinal absorption rate constant (*k_a_*, min^−1^). Compounds exhibiting %HIA > 70% were classified as well absorbed, those with %HIA < 30%—poorly absorbed. Values in the range of 30–70% represented moderate absorption [41,50].

The Blood-Brain Penetration module predicted compounds’ brain penetration potential. Evaluation was based on predicted brain/plasma equilibration rate (log(PS*fu,brain)) and steady-state brain/plasma distribution ratio (log*BB*) [43]. According to the values of CNS Access Score (Score = log(PS*fu,brain) + log*BB*) compounds were denoted as non-penetrants (Score ≤ −3.5), weak penetrants (−3.5 ≤ Score ≤ −3.0) and penetrants (Score ˃ −3) [41].

The Plasma Protein Binding module predicted plasma protein bound fraction (%PPB) and the equilibrium binding constant to serum albumin of a compound (log*K_a_^HSA^*). The predictive models of %PPB and log*K_a_^HSA^* were derived using the Global, Adjusted Locally According to Similarity (GALAS) modelling methodology [51]. Compounds exhibiting %PPB ≤ 10% were classified as not bound those with %PPB > 90% as extensively bound. Values in the range 10% < %PPB ≤ 40% and 40% < %PPB ≤ 80% were characteristic for compounds weakly and moderately bounded, respectively. Values in the ranges 80% < %PPB ≤90% referred to strongly bounded compounds [41].

In acute Toxicity Module compounds were assigned to one of the five “Oral Acute Toxicity Hazard Categories” according to the numeric criteria expressed as LD_50_ (mg/kg) (oral administration to rats). Categories were defined by the Organization for Economic Cooperation and Development (OECD) Guide to The Globally Harmonized System of Classification and Labeling of Chemicals (GHS): V—LD_50_ 2000–5000 mg/kg (may be harmful if swallowed), IV—300–2000 mg/kg (harmful if swallowed), III—50–300 mg/kg (toxic if swallowed), II—5–50 mg/kg (fatal if swallowed), I < 5 mg/kg (fatal if swallowed) [41,46].

All predictions were supported by Reliability Index (RI) values that represented a quantitative evaluation of prediction confidence. High RI showed that the calculated value was likely to be accurate, while low RI indicated that no similar compounds with consistent data were present in the training set and the structure may be outside the structural space covered by the training set that was used to build the algorithm [41].

## 4. Conclusions

The present paper reports the synthesis of new symmetrical polyamine conjugates with bicyclic quinazoline **4**–**6**, naphthalene **11**, quinoline **12**, coumarin **13** and indole **14** moieties tethered by 1,4-bis(3-aminopropyl)piperazine (**a**), 4,9-dioxa-1,12-dodecanediamine (**b**), 3,3′-diamino-*N*-methyl-dipropylamine (**c**) and bis(3-aminopropyl)amine (**d**).

Although in comparison to doxorubicin (IC_50_: 1.51 μM, MCF–7; 1.22, PC–3; 0.58, DU–145) [52] the newly synthesized compounds exhibited lower anticancer activity against the aforementioned cell lines, bis(naphthalene-2-carboxamides) derivatives, e.g., **11c** and **11d**, demonstrated relatively promising antiproliferative properties with IC_50_ values in the 6.00–23.30 μM range. Moreover, they caused ds-oligonucleotide melting temperature increments and converted relaxed plasmid DNA into supercoiled DNA, what provides evidence that they bind to DNA in an intercalative manner. Therefore, it can be postulated that the presence of a naphthalene moiety together with 3,3′-diamino-*N*-methyldipropylamine (**c**) or bis(3-aminopropyl)amine (**d**) as linkers is crucial for the assumed binding mode. These linkers are also optimal for biological activity. Furthermore, it is important to mention that removing the methyl group from the central nitrogen atom of 3,3′-diamino-*N*-methyldipropylamine **11c** slightly enhanced the cytotoxic activity and improved the ds-DNA binding parameters (Table 1 and Table 2 and Figure 4). Taking into account the preliminary in silico ADMET screening of the most active compounds, it can be noted that compounds **4a**, **11c**, **11d**, **12d**, **14c**, **14d** showed favourable *drug-like* properties according to *Lipinski’s Rule of Five*. In conclusion, interesting biological features found for bis(naphthalene-2-carboxamide) with 3,3′-diamino-*N*-methyldipropyl-amine **11c** or bis(3-aminopropyl)amine **11d** as linkers provide a promising basis for further development of potential anticancer drugs.

## Figures and Tables

**Table 1:** Cytotoxicity of polyamine conjugates with bicyclic systems towards prostate cancer cells and breast cancer cells.

**Entry**	**Viability Rate %**									
	**PC–3**									
	**5 µM**	**10 µM**	**15 µM**	**20 µM**	**25 µM**	**30 µM**	**35 µM**	**40 µM**	**50 µM**	**^1^** **IC_50_ µM**
**4a**	95.94 ± 2.25	98.80 ± 1.39	91.90 ± 1.93	69.92 ± 3.33	63.74 ± 2.85	46.91 ± 1.61	32.62 ± 1.33	21.61 ± 1.32	11.08 ± 0.57	28.24
**11c**	…	96.10 ± 2.18	95.82 ± 1.2	73.53 ± 3.9	34.14 ± 2.5	23.60± 4.4	6.16 ± 0.60	4.73 ± 0.24	3.45 ± 0.14	23.30
**11d**	96.69 ± 0.77	97.05 ± 0.58	58.49 ± 6.00	10.70 ± 5.02	3.44 ± 0.24	3.68 ± 0.22	3.50 ± 0.24	3.04 ± 0.21	2.93 ± 0.21	22.57
**12d**	95.31 ± 3.97	92.83 ± 1.93	81.70 ± 7.40	80.66 ± 5.28	72.56 ± 5.78	65.12 ± 3.77	58.44 ± 3.44	59.54 ± 5.09	48.39 ± 1.96	48.08
**14c**	95.62 ± 0.91	92.34 ± 3.30	89.42 ± 5.22	99.90 ± 5.57	90.33 ± 4.90	47.94 ± 10.29	62.48 ± 5.82	45.69 ± 4.25	9.57 ± 2.74	35.72
**14d**	92.66 ± 5.82	109.44 ± 4.12	113.08 ± 7.15	87.05 ± 3.24	52.95 ± 1.97	38.51 ± 1.58	25.82 ± 1.61	19.12 ± 0.91	5.21 ± 1.41	27.59
**Entry**	**Viability Rate %**									
	**DU–145**									
	**5 µM**	**10 µM**	**15 µM**	**20 µM**	**25 µM**	**30 µM**	**35 µM**	**40 µM**	**50 µM**	**^1^** **IC_50_ µM**
**4a**	91.23 ± 3.47	100.58 ± 3.57	93.26 ± 2.16	91.12 ± 3.24	86.85 ± 1.78	78.77 ± 2.71	51.02 ± 6.52	54.00 ± 2.21	28.24 ± 1.85	43.63
**11c**	94.90 ± 2.95	76.11 ± 4.76	35.16 ± 1.53	11.99 ± 0.91	3.07 ± 0.09	3.02 ± 0.16	3.16 ± 0.16	3.11 ± 0.09	3.25 ± 0.14	12.96
**11d**	80.23 ± 2.50	30.43 ± 1.33	4.72 ± 0.10	3.45 ± 0.22	3.52 ± 0.15	3.58 ± 0.13	3.63 ± 0.25	3.61 ± 0.13	3.40 ± 0.20	7.63
**12d**	80.81 ± 2.32	77.77 ± 1.63	73.62 ± 2.78	72.97 ± 2.97	65.67 ± 2.49	59.84 ± 3.21	51.32 ± 3.18	42.75 ± 3.06	33.24 ± 4.39	42.63
**14c**	95.04 ± 2.49	80.02 ± 1.82	60.43 ± 1.77	30.57 ± 1.19	7.28 ± 0.68	4.82 ± 0.38	4.19 ± 0.39	3.78 ± 0.36	3.77 ± 0.39	15.86
**14d**	93.25 ± 1.24	78.47 ± 1.67	57.33 ± 1.36	36.78 ± 0.43	24.58 ± 0.80	18.89 ± 0.57	12.37 ± 0.27	8.16 ± 0.29	5.07 ± 0.12	16.46
**Entry**	**Viability Rate %**									
	**MCF–7**									
	**5 µM**	**10 µM**	**15 µM**	**20 µM**	**25 µM**	**30 µM**	**35 M**	**40 M**	**50 M**	**^1^** **IC_50_ µM**
**4a**	75.91 ± 1.39	88.72 ± 2.36	78.00 ± 3.89	31.76 ± 8.36	12.79 ± 0.53	14.51 ± 0.63	12.95 ± 0.58	11.32 ± 1.02	7.38 ± 0.53	17.95
**11c**	74.01 ± 3.18	27.88 ± 7.04	22.58 ± 1.05	11.25 ± 0.63	5.88 ± 0.33	4.86 ± 0.24	4.56 ± 0.14	4.73 ± 0.35	4.98 ± 0.19	7.48
**11d**	65.16 ± 4.29	13.49 ± 1.31	5.08 ± 0.26	4.97 ± 0.32	5.14 ± 0.36	5.44 ± 0.41	5.74 ± 0.20	5.74 ± 0.39	6.58 ± 0.28	6.00
**12d**	61.13 ± 3.10	68.18 ± 2.27	63.21 ± 2.57	62.06 ± 0.95	49.05 ± 3.46	53.31 ± 2.23	42.92 ± 1.78	42.54 ± 1.93	29.53 ± 1.51	25.45
**14c**	149.95 ± 13.45	106.03 ± 19.03	46.02 ± 5.56	22.48 ± 2.32	11.71 ± 1.87	8.66 ± 1.17	7.96 ± 0.99	7.83 ± 0.92	8.23 ± 0.98	15.51
**14d**	80.29 ± 1.26	79.63 ± 1.68	58.73 ± 3.57	45.53 ± 2.32	29.16 ± 1.13	14.52 ± 1.33	9.43 ± 1.07	6.61 ± 0.32	5.26 ± 0.23	16.91

^1^ IC_50_ is the drug concentration effective in inhibiting 50% of the cell viability measured by WST-1 cell proliferation assay after 48 h exposure. GraphPad Prism was employed to produce dose-response curves by performing nonlinear regression analysis. The viability of the treated cells was normalized to the viability of the untreated (control) cells, and cell viability fractions were plotted versus drug concentrations in the logarithmic scale. IC_50_ values were reported as mean values.

**Table 2:** Influence of examined compounds on ds–DNA thermal stability.

Additive	Oligonucleotide	Melting Temperature, *T*_m_ (°C)
**None (negative control)**	ds–DNA	61.69 ± 0.58
**4a**	ds–DNA	61.67 ± 0.56
**11c**	ds–DNA	65.10 ± 0.11
**11d**	ds–DNA	67.52 ± 0.72
**12d**	ds–DNA	61.02 ± 0
**14c**	ds–DNA	61.34 ± 1.18
**14d**	ds–DNA	61.02 ± 1.73
**9-AA (positive control)**	ds–DNA	70.08 ± 1.08

**Table 3:** Drug-likeness parameters for the biologically active compounds.

Entry	*Drug-likeness*			
	^1^ HBD	^2^ HBA	^3^ M_w_	^4^ log*P*
**4a**	0	8	486.61	2.18
**11c**	2	5	453.58	4.17
**11d**	3	5	439.55	4.37
**12d**	3	7	441.53	2.34
**14c**	4	7	431.53	3.6
**14d**	5	7	417.5	3.8

^1^ HBD—number of hydrogen bond donors; ^2^ HBA—number of hydrogen bond acceptors; ^3^ M_w_—molecular weight; ^4^ log*P—*the logarithm value of octanol-water partition coefficient.

**Table 4:** In silico ADMET parameters for the biologically active compounds.

Computed ADMET Parameters	4a	11c	11d	12d	14c	14d
**^1^ %HIA**	100	100	100	99.02	99.57	94.54
**^2^*Pe*, 10^−4^ cm/s**	6.14	6.73	6.11	1.77	2.23	0.97
**^3^*k_a_*, min^−1^**	0.04	0.05	0.04	0.01	0.02	0.01
**^4^ log*PS***	−2.17	−1.65	−1.93	−2.93	−2.73	−2.99
**^5^ log*BB***	0.1	−0.22	−0.31	−0.93	−0.08	−0.08
**^6^** **fu, brain**	0.27	0.01	0.02	0.19	0.08	0.08
**^7^** log(PS*fu, brain)	−2.73	−3.58	−3.65	−3.64	−3.84	−4.06
**^8^** **%PPB**	65.87	99.38	98.91	97.74	93.48	92.91
**^9^** **log *K_a_^HSA^***	3.48	5.04	4.98	4.65	4.2	4.23
**^10^** **V (L/kg)**	7.51	6.53	6.78	4.63	6.13	6.00
**^11^** **LD_50_**	190	1600	1600	850	430	420

^1^ %HIA—the maximum achievable extent of human intestinal absorption; ^2^ *P_e_*, 10^−4^ cm/s—effective jejunal permeability coefficients at pH 6.5; ^3^ *k*_a_—absorption rate constants (min^−1^); ^4^ log*PS*—the rate of brain penetration; ^5^ log*BB*—extent of brain penetration; ^6^ fu, brain—fraction unbound in brain tissue; ^7^ log(PS*fu, brain)—brain/plasma equilibration rate; ^8^ %PPB—the cumulative percentage of the analyzed compound bound to human plasma proteins; ^9^ log *K_a_^HSA^*—the drug’s affinity constant to human serum albumin; ^10^ V (L/kg)—calculated apparent volume of distribution of a compound; ^11^ LD_50_ (mg/kg)—acute toxicity for rat after oral administration.
